# CLaSSiNet: A Computational Framework for High-Resolution Classification and Spatial Mapping of Heterogeneous Biological Network Architectures

**DOI:** 10.1021/jacsau.5c01775

**Published:** 2026-03-27

**Authors:** Yuan Tao, Ruobo Zhou

**Affiliations:** † Department of Chemistry, 311285 The Pennsylvania State University, University Park, Pennsylvania 16802, United States; ‡ The Huck Institutes of Life Sciences, The Pennsylvania State University, University Park, Pennsylvania 16802, United States; § Department of Biochemistry and Molecular Biology, The Pennsylvania State University, University Park, Pennsylvania 16802, United States; ∥ Department of Biomedical Engineering, The Pennsylvania State University, University Park, Pennsylvania 16802, United States

**Keywords:** Super-Resolution Imaging, Single-Molecule Localization Microscopy (SMLM), Membrane-Associated Periodic Skeleton (MPS), Image Analysis, Cytoskeleton, Structural Organization Classification, Network Topology

## Abstract

Quantitative analysis of network-like biological molecular architectures, such as cytoskeletal networks, remains a fundamental challenge in super-resolution fluorescence imaging because single-molecule localization microscopy (SMLM) typically produces sparse and discontinuous localization patterns arising from stochastic labeling, incomplete probe occupancy, and structural deformation of biological networks. These factors obscure the underlying connectivity, periodicity, and symmetry, limiting the ability of existing analysis methods to resolve higher-order organization. Here, we report the Classifier of Super-resolution Structural Networks (**CLaSSiNet**), a conceptually novel computational framework that overcomes these sparsity and heterogeneity constraints. By integrating connectivity, 1D periodicity, and 2D regularity classifiers through newly developed algorithms, CLaSSiNet sensitively captures the organizational signatures of imaged networks to automatically segment and map networks with an unprecedented resolution (∼256 nm, reaching the diffraction limit of light). CLaSSiNet uniquely resolves four distinct organizational states (1D periodic, 2D polygonal, disordered, and non-network), providing a robust platform for analyzing SMLM data sets regardless of labeling chemistry. Using CLaSSiNet, we achieve the first spatially resolved, quantitative mapping of organizational heterogeneity in the actin-spectrin membrane-associated periodic skeleton (MPS), a conserved cytoskeletal network located underneath the plasma membrane of animal cells. This analysis reveals previously unrecognized organizational principles for these MPS networks, with ordered 1D and 2D networks enriched at cell edges and junctions, while non-network states dominate the cell body. Furthermore, we uncover a mechanical coupling principle wherein actin stress fibers bias the symmetry and orientation of nearby spectrin lattices, indicating bidirectional coordination between contractile actin bundles and periodic MPS networks. Comparative analysis across diverse cell types highlights the cell-specific “tuning” of these supramolecular design rules. Broadly, CLaSSiNet establishes a principled computational framework for dissecting the nanoscale design rules of complex molecular networks, offering a robust methodology applicable to the wider study of hierarchical biological and bioinspired architectures.

## Graphical abstract

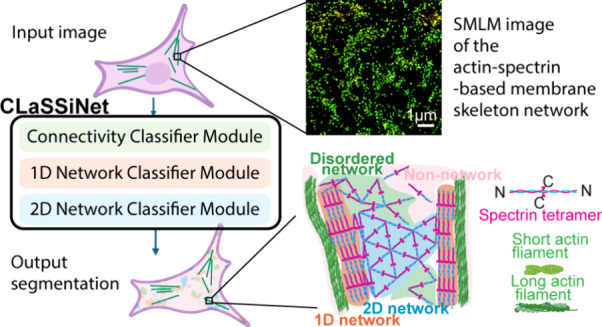

## Introduction

Super-resolution fluorescence microscopy, specifically single-molecule localization microscopy (SMLM), has revolutionized our ability to visualize the nanoscale organization of cellular structures, revealing a rich diversity of network-like architectures that underlie many fundamental biological processes.^1–3^ These networks can often be abstracted into “nodes” (e.g., protein complexes, filament junctions) physically connected by “links” (e.g., cytoskeletal filaments, membrane tubules), spanning a wide range of dimensionalities, geometries, and degrees of structural regularity (Figure 1A). For instance, the membrane-associated periodic skeleton (MPS) in neurons predominantly forms a 1D periodic network in axons, where short actin filaments (nodes) are regularly spaced at ∼190 nm intervals and connected by spectrin tetramers^4^ (links of comparable length; Figure 1B). In somas and dendrites of neurons, the same MPS components can give rise to a more variable 2D polygonal network with less regular spacing and orientation^5,6^ (Figure 1B). The erythrocyte membrane skeleton represents a similar 2D polygonal network, composed of actin nodes and non-neuronal spectrin isoforms that generate average link lengths of 70–80 nm.^7^ The sarcomere, the repeating unit of muscle fibers responsible for muscle contraction, exemplifies a 1D periodic contractile network, where actin and myosin filaments are precisely arranged between Z-discs spaced roughly 2.2 μm apart.^8^ In contrast, networks such as the endoplasmic reticulum (ER) tubular system^9,10^ and tight junction strands^11–13^ in epithelial layers form more irregular polygonal architectures characterized by dynamic remodeling and substantial variability in link geometry, with link lengths often ranging from a few hundred nanometers to several micrometers. These diverse node-link networks play essential roles in supporting cellular architecture, enabling mechanical force transmission, organizing intracellular compartments, regulating molecular transport, and coordinating localized signaling events.^13–16^ Despite their critical roles in force transmission, intracellular organization, and signaling, there remains a lack of robust, automated computational tools to reliably identify, quantify, and classify such structurally diverse cellular networks, particularly in large-scale or noisy super-resolution imaging data sets.^17^

**Figure 1:**
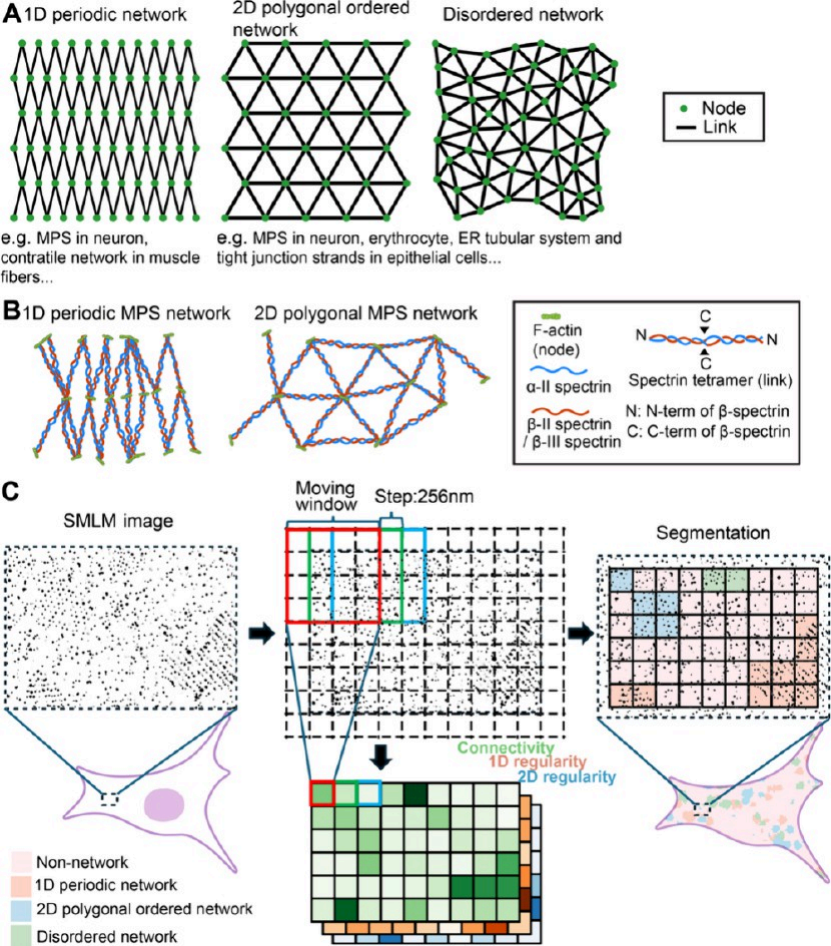
Example forms of network-like cellular architectures underlying fundamental biological processes and overview of the CLaSSiNet pipeline for their quantification and segmentation. (A) Schematics illustrating three distinct organizational states of network-like cellular architectures: one-dimensional (1D) periodic network, two-dimensional (2D) polygonal ordered network, and disordered network. (B) Schematics illustrating the organizations of 1D periodic MPS network and 2D polygonal MPS network. (C) Schematic overview of our developed CLaSSiNet pipeline for quantitative analysis and segmentation of super-resolution (SMLM) images of cellular networks. In each SMLM image, the cellular area containing signals is divided into a grid of 256 × 256 nm pixels. A 4 × 4-pixel moving window scans across the entire cellular area, and three classifier modules are applied within the moving window to compute likelihood scores corresponding to distinct organizational states. Each classifier module generates a heatmap representing one of three quantitative measures: connectivity (network-forming tendency), 1D regularity, and 2D regularity. The resulting heatmaps are then thresholded, binarized, and integrated to produce a segmentation map at 256 nm resolution, dividing the cellular area into four regions: 1D periodic, 2D polygonal ordered, disordered, and non-network regions.

Here, we present **C**lassifier of **S**uper-resolution **S**tructural **N**etworks (**CLaSSiNet**), a modular pipeline for the quantitative analysis of network-like cellular architectures from SMLM imaging data (Figure 1C). CLaSSiNet first identifies the cellular area in an SMLM image and then applies three classifier modules that segment the cellular area into four organizational subregions: regions primarily containing 1D periodic network, regions primarily containing 2D polygonal ordered network, regions primarily containing disordered network, and regions primarily containing non-network state. To enable high-resolution segmentation, the cellular area is divided into a grid with pixels of 256 × 256 nm. A moving window of 4 × 4 pixels (step size: 1 pixel) is then used as the analysis unit to scan across the entire cell area in the SMLM image. Each classifier module generates a heatmap representing one of three quantitative measures: connectivity (network-forming tendency), 1D periodicity, or 2D regularity, respectively. These heatmaps are subsequently thresholded, binarized, and integrated to generate a segmentation map with 256 nm resolution for each analyzed SMLM image.

Using the MPS, an actin-spectrin-based membrane skeleton present beneath the plasma membrane in many animal cell types^18–22^ and known to support essential cellular functions such as membrane protein anchoring, regulation of cellular mechanics, endocytosis, and membrane-associated signaling,^23–26^ as a model system (Figure 1B), we applied CLaSSiNet to compare MPS organization in three different cell types: epithelial U2OS cells, fibroblast 3T3 cells, and neurons, uncovering previously unappreciated cell-type-specific organizational patterns. To further demonstrate the biological insights enabled by CLaSSiNet-based high-resolution network state segmentation, we analyzed how the MPS is organized around actin stress fibers, another major cytoskeletal structure formed at the adherent cell surface. We discovered that the likelihood of forming 1D periodic networks increases near stress fibers and that the axis of MPS periodicity within these 1D-network-enriched regions is preferentially oriented perpendicular to the stress fibers. Together, these findings establish CLaSSiNet as a versatile and quantitative framework for dissecting nanoscale network organizational states across different cell types and subcellular contexts. By providing a systematic means to distinguish and compare diverse network architectures, CLaSSiNet enables the discovery of previously unknown structural principles that may govern how cytoskeletal and membrane-associated networks adapt to specific cellular environments. Beyond the MPS, CLaSSiNet offers a broadly applicable strategy for probing the hierarchical organization of diverse node-link networks, providing a robust framework to uncover the structural design rules that couple nanoscale patterning to complex cellular functions.

## Results

### Connectivity Classifier Module for Quantifying the Network-Forming Tendency

In SMLM imaging, it is often not possible to directly visualize an entire biological network including all nodes and their connecting links. Instead, immunofluorescence (IF) labeling typically reveals only the antigen positions at which dye-conjugated antibodies bind. Therefore, reconstructing the node-link network of the biological networks from an SMLM image of a single cell requires a method that can infer the underlying connectivity based on the visualized nodes and quantify local structural variations in network organization.

In this study, we used the actin–spectrin-based MPS as a model system for biological network analysis. The MPS is a ubiquitous and evolutionarily conserved cytoskeletal network underlying the plasma membrane of many cell types and is composed primarily of short actin filaments and actin-binding proteins, termed actin “nodes”, which are connected by spectrin tetramers that serve as “links”. The MPS can adopt either a one-dimensional (1D) periodic network, a two-dimensional (2D) polygonal ordered network, or a disordered network, each covering certain portions of the plasma membrane (Figure 1A,B). IF-compatible antibodies have been developed to target the actin nodes, including antibodies that recognize short actin filaments or the N-termini of βII- or βIII-spectrin in cultured neurons.^20^ These antibodies enable the acquisition of experimental SMLM images of actin node distributions (termed SMLM node images)

Because experimental SMLM node images do not provide ground truth network structures, which makes quantitative assessment of any network classifier impossible, we first aimed to establish a simulation method that generates node distributions with defined ground truth for each distinct organizational state of the MPS. Guided by experimentally measured SMLM node distributions from neurons,^20^ the simulation begins with idealized 1D periodic or 2D polygonal ordered networks (Figure 1A) and introduces parametrized perturbations, including missed detections, false-positive detections, and lateral positional deviations of nodes. These introduced perturbations were tuned such that the simulated SMLM images reproduced the experimentally observed nearest-neighbor distance distributions for each organizational state. Using this approach, we generated simulated SMLM images of actin nodes arranged in three organizational states: (1) 1D periodic networks, (2) 2D polygonal ordered networks, and (3) randomly distributed nodes representing non-network regions (Figure S1). This simulation framework allows the generation of SMLM node images containing embedded “islands” of nodes arranged in any organizational state, such as 1D periodic, 2D polygonal ordered, on a background that can also adopt any combination of organizational states, providing a versatile and controlled data set for assessing the performance of our classifier modules in segmenting SMLM images into subregions with distinct MPS organizational states.

Next, we developed our first classifier module, which reconstructs the node-link network of the MPS from SMLM node-only images and classifies the cellular area into network and non-network regions. To reconstruct the underlying network from SMLM node-only images, we considered two network reconstruction algorithms for the simulated SMLM node images (Figure 2A; Figure S2A,B). (1) Range-based network reconstruction: nodes within a predefined distance range around the average link length (e.g., 190 ± 30 nm for MPS) were connected, and one of any intersecting links was randomly removed. (2) Delaunay-based network reconstruction: a Delaunay triangulation was generated from the node image, and links outside the predefined distance range (190 ± 30 nm) were removed except when only one link in a triangle was out of range and the other two links of the same triangle were within the range. To quantify the reconstructed network’s connectivity, defined as the extent of interconnectedness per unit area, we used three measures based on the reconstructed network: (i) **link count**, number of links the reconstructed network contains per unit area, (ii) **triangle count**, number of triangles the reconstructed network contains per unit area (Triangles are defined as the smallest closed polygonal units in the reconstructed network, formed when three links connect neighboring nodes to enclose an area), and (iii) **triangle area sum**, total triangle area per unit area. Together, these produced six candidate reconstruction–quantification pipelines (two reconstruction algorithms × three connectivity measures). Using the aforementioned moving-window approach to divide each SMLM image into grids (Figures 1C and 2A), connectivity heatmaps can be generated across the entire cellular area in the SMLM image.

**Figure 2:**
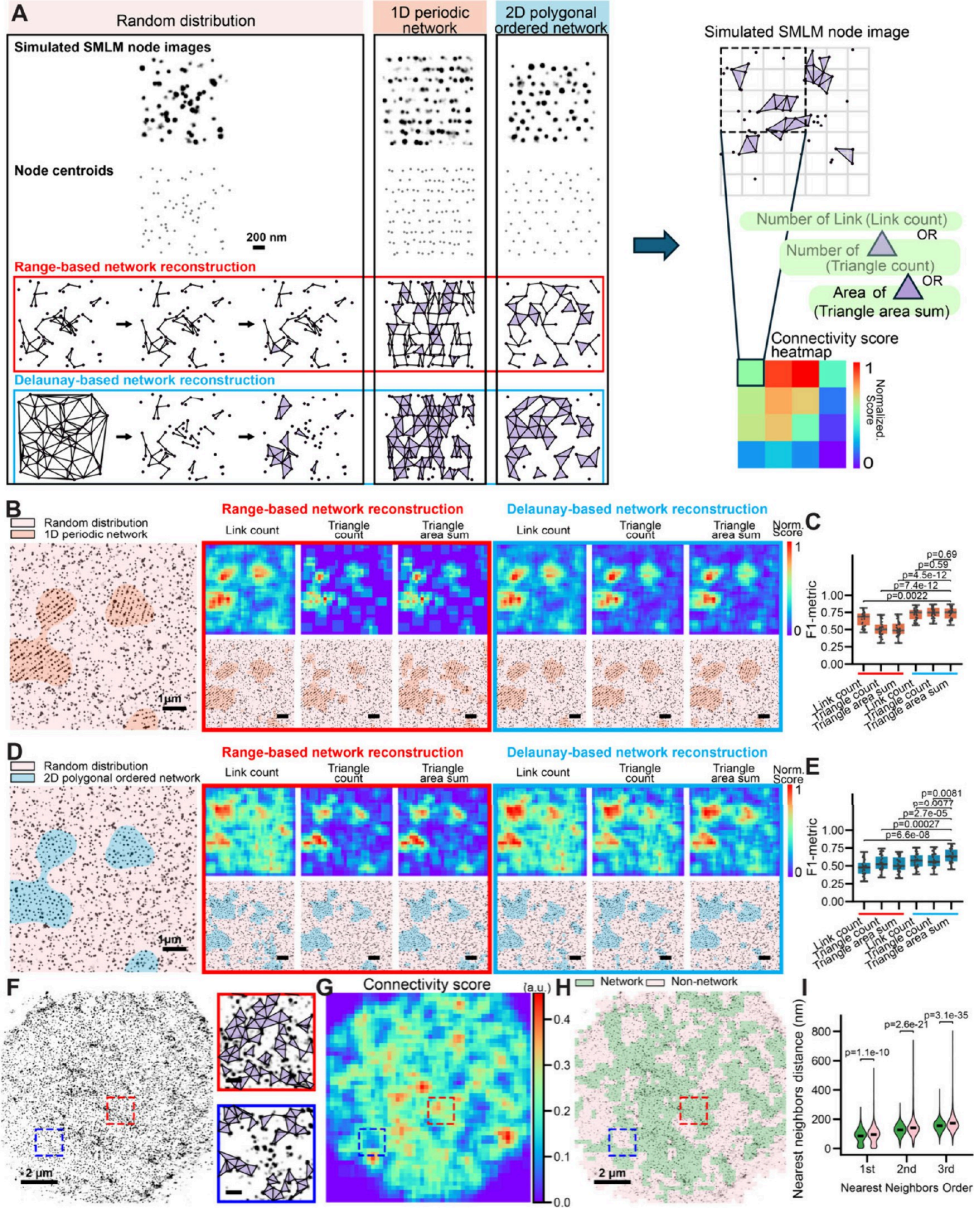
Connectivity Classifier Module for quantification of network-forming tendency and segmentation of network versus non-network cellular regions. (A) Top left: representative simulated SMLM node images showing random, 1D periodic, and 2D polygonal distributions. Middle: reconstructed networks generated from the three simulated images using range-based network reconstruction. Bottom left: reconstructed networks obtained using Delaunay-based network reconstruction. The reconstruction steps are shown only for the simulated SMLM node image with random distribution. Right: Schematic illustrating three quantitative measures of connectivity (network-forming tendency), including link count, triangle count, and triangle area sum, and the generation of the corresponding connectivity heatmap. As shown in Figure 1C, each reconstructed network image was divided into 256 × 256 nm grids, and a 4 × 4-pixel moving window (step size: 1 pixel) was applied to compute the three measures and generate a connectivity heatmap. (B) Left: representative simulated SMLM node image containing embedded islands of nodes arranged in a 1D periodic network, surrounded by randomly distributed nodes of equal density. Right: connectivity heatmaps (top) and segmented images (bottom) generated for the simulated SMLM image on the left, using six reconstruction-quantification pipelines (two reconstruction algorithms × three connectivity measures). Scale bars: 1 μm. (C) Boxplots of F1-metrics quantifying the segmentation performance in distinguishing 1D periodic network regions from non-network (randomly distributed) regions. (D,E) Same as (B,C) but for simulated SMLM node images containing embedded islands of nodes arranged in a 2D polygonal network surrounded by randomly distributed nodes of equal density. (F) Left: representative experimental SMLM image of βIII-spectrin (immunolabeled at its N-terminus). Right: magnified views of the two boxed regions showing the reconstructed network. Scale bars: 2 μm. (G,H) Connectivity heatmap (G) and segmented image (H) generated for the SMLM image in (F). (I) Violin plots of the first, second, and third nearest-neighbor distances (NNDs), determined for network versus non-network regions. Center lines indicate the median. *p*-values were calculated using a two-sided unpaired Student’s *t*-test.

To assess performance, we simulated SMLM node images containing embedded “islands” of nodes arranged in either a 1D periodic (Figure 2B) or 2D polygonal (Figure 2D) network, surrounded by nodes of equal density but random distribution. An effective reconstruction–quantification pipeline should sensitively detect the network-organized islands and distinguish them from the random background, which is expected to primarily consist of non-network nodes. Connectivity heatmaps generated from the six pipelines all successfully outlined the islands, albeit with varying levels of error. By applying thresholds for each connectivity measure (determined at a 5% false-positive rate, Figure S2C,D), we converted the heatmaps into binary segmentation maps of network versus non-network regions (Figure 2B,D). Performance was quantified using the F1 metric,^27^ which is the harmonic mean of precision and recall of a classification model (Figure 2C,E). F1 metric analysis revealed that Delaunay-based network reconstruction combined with any of the three measures achieved higher F1 metrics than range-based network reconstruction for distinguishing 1D periodic networks from random distributions. For 2D networks, the best performance was achieved with Delaunay-based network reconstruction combined with the triangle area sum. Based on these results, we selected Delaunay-based network reconstruction with triangle area sum as the optimal pipeline, which we referred to as the Connectivity Classifier Module, as it most sensitively distinguished both 1D and 2D networked nodes from random (i.e., non-network) nodes.

To evaluate the sensitivity and robustness of the Connectivity Classifier Module to experimental noise, we applied it to simulated SMLM images of 1D and 2D networks with controlled perturbations. Five common noise sources were introduced: lateral positional deviations of node localization clusters, added false-positive nodes (e.g., fluorescent debris), missed node detections (e.g., low labeling efficiency), background localization noise (i.e., randomly distributed, nonclustered localizations), and variation in per-node localization number (e.g., differing numbers of SMLM movie frames collected). For each condition, we quantified F1 metrics to assess the classification performance. For the three node-level perturbations (lateral deviations, added false positives, and missed detections), the F1 metrics decreased progressively with increasing noise. The classifier remained highly robust to added false positive nodes, whereas lateral positional deviations of nodes and missed node detections produced a stronger degradation in performance (Figure S2E,F). For the two localization-level perturbations, increasing background localization noise led to a gradual decline in the F1 metric, whereas reducing per-node localization number resulted in only a modest decrease in performance. Notably, even under the least robust condition (i.e., missed node detections), the F1 metric declined gradually and remained >0.5 up to a 40–50% miss rate. Although an F1 metric of 0.5 usually represents the lower bound of acceptable classification performance, this result indicates that the Connectivity Classifier Module continues to extract meaningful network structure, even under substantial signal degradation.

Finally, we applied our Connectivity Classifier Module to experimental SMLM images of cultured hippocampal neurons immunostained for the N-terminus of βIII-spectrin, labeling the actin nodes (Figure 2F). The resulting binary segmentation maps showed that regions classified as “network” corresponded to areas with higher reconstructed link density (Figure 2G,H). To further characterize network organization, we performed nearest-neighbor distance (NND) analysis, which quantifies the distances between each node and its closest neighboring nodes within the network. Specifically, we calculated the first, second, and third NNDs for all nodes, where the first NND represents the distance to the closest neighboring node, the second NND to the next closest, and the third NND to the third closest neighbor. This analysis revealed that non-network regions exhibited significantly longer first, second, and third NNDs compared to network regions, reflecting the lower local node density and lack of organized connectivity in non-network regions, whereas network regions maintained shorter and more regular node spacing consistent with structured MPS organization (Figure 2I).

### 1D Network Classifier Module to Quantify the Regularity of 1D Periodic Networks

After segmenting SMLM node images into network and non-network regions using the Connectivity Classifier Module, we next sought to specifically identify and characterize 1D periodic networks, such as the 190 nm MPS organization observed in neuronal axons and dendrites.^20,28^ To achieve this, we developed a two-dimensional Fast Fourier Transform (2D FFT)-based 1D Network Classifier Module that quantifies the degree of 1D periodic order in SMLM node images (Figure 3A).

**Figure 3:**
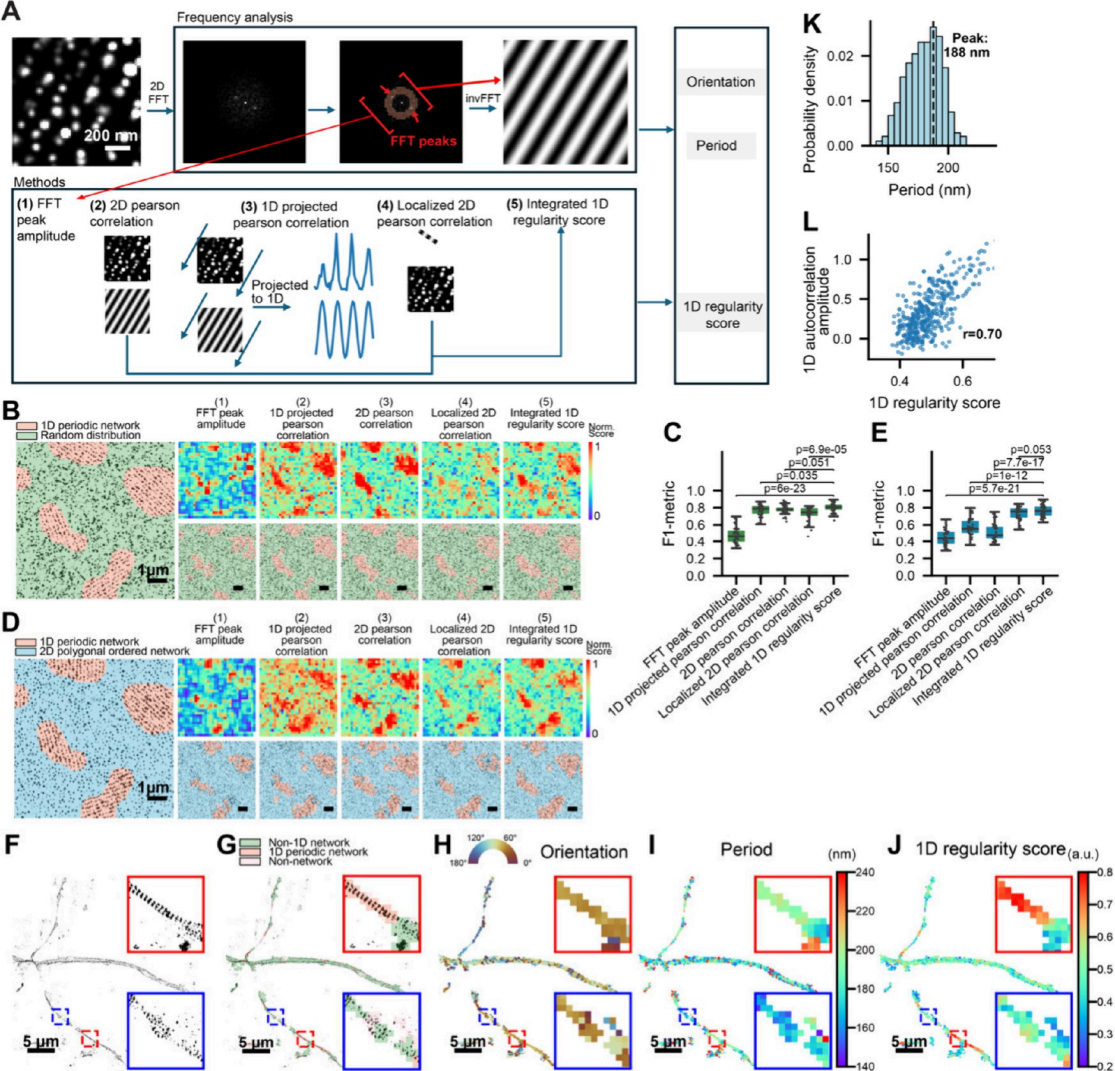
1D Network Classifier Module for quantification of 1D regularity and segmentation of 1D network versus non-1D network regions. (A) Schematic overview of the candidate quantification methods used to optimize the 1D Network Classifier Module. Within each moving window (1024 nm × 1024 nm), a 2D FFT identifies dominant frequency peaks within the annular region corresponding to real-space periods of 190 ± 30 nm, and an inverse FFT generates a grating image representing the best-fitting 1D orientation and period. Five candidate 1D regularity scores are illustrated: FFT peak amplitude, 2D Pearson correlation, 1D projected Pearson correlation, localized 2D Pearson correlation, and the integrated 1D regularity score. See main text for full definitions. (B) Left: Representative simulated SMLM node image containing embedded islands of nodes arranged in a 1D periodic network, surrounded by randomly distributed nodes of equal density. Right: 1D regularity score heatmaps (top) and segmented images (bottom) generated for the simulated SMLM image on the left using the five 1D regularity scores described in (A). Scale bars: 1 μm. (C) Boxplots of F1-metrics quantifying segmentation performance in distinguishing 1D periodic network regions from random node distributions. (D,E) Same as (B,C), but for simulated SMLM node images containing embedded islands of nodes arranged in a 1D periodic network, surrounded by nodes forming a 2D polygonal network of equal density. Boxplots show the median and interquartile range (first and third quartiles); whiskers denote the minimum and maximum values excluding outliers. *p*-values were calculated using a two-sided unpaired Student’s *t*-test. (F) Experimental SMLM (STORM) image of βIII-spectrin in dendrites. Top right inset: magnified view of the boxed region showing high 1D regularity scores. (G–J) Segmented image (G), orientation heatmap (H), period heatmap (I), and 1D regularity score heatmap (J) generated for the SMLM image in (F). Scale bars: 5 μm. (K) Distribution of 1D periods obtained from the segmented 1D periodic regions identified in (J), showing a peak at ∼188 nm. (L) Scatter plot showing a strong positive correlation (*r* = 0.70) between 1D autocorrelation amplitudes determined by traditional 1D autocorrelation analysis and the 1D regularity scores determined by the 1D Network Classifier Module, calculated from the same set of experimental SMLM images.

This module used the aforementioned moving-window approach to divide each SMLM image into grids (Figure 1C). For each moving window (1024 × 1024 nm, with moving step 256 nm), a 2D FFT was performed to convert the spatial image into the frequency domain. In the resulting 2D frequency domain image, the pair of peaks with the highest amplitudes within a donut-shaped region corresponding to a real-space period of 190 ± 30 nm (the average period previously determined for 1D MPS) was identified as the dominant frequency peaks. By retaining only these two peaks and removing all other frequency components, an inverse FFT (invFFT) was applied to generate a real-space grating image representing the best-fitting orientation and period of the potential 1D network within the moving window. To quantify the degree of 1D periodic order, we calculated five candidate 1D regularity scores within the moving window: (1) FFT peak amplitude, the amplitude of the two dominant frequency peaks in the 2D frequency domain image; (2) 2D Pearson correlation, the Pearson correlation coefficient between the raw SMLM image and the invFFT-generated grating image; (3) 1D projected Pearson correlation, the Pearson correlation coefficient between 1D signal profiles obtained by projecting both images in (2) along the grating axis; (4) localized 2D Pearson correlation, similar to (2), but the correlation is calculated between the raw SMLM image and a cropped, elongated grating image to account for cases where the 1D periodic network occupies only a subregion within the moving window; and (5) integrated 1D regularity score, a combined metric that mathematically integrates measures (2) and (4) to account for both large 1D network islands spanning the full window and smaller local 1D regions.

As the moving window scans across the SMLM node image, each 1D regularity score generates a spatial heatmap of the 1D periodicity. In addition, the orientation and period associated with each window can be directly derived from the spatial coordinates of the dominant frequency peaks.

To compare the performance of these five 1D regularity scores, we simulated SMLM node images containing embedded islands with 1D periodic distributions, surrounded by either randomly distributed nodes of equal density or nodes forming 2D polygonal ordered networks (Figure 3B,C). Each 1D regularity score produced a corresponding heatmap of 1D periodic order. Because each heatmap pixel (256 × 256 nm) is smaller than the typical size of most 1D network islands, and the orientation and period values of adjacent pixels vary only gradually, we incorporated the score, orientation, and period into a segmentation method based on a seed region growing algorithm (Figure S3A,B). Specifically, a stringent initial threshold—corresponding to a 1% false-positive rate (Figure S3C)—was applied to the 1D regularity score heatmap to identify high-confidence “seed” pixels. These seed regions were then expanded by iteratively incorporating adjacent pixels that satisfied two criteria: (i) their 1D regularity score exceeded a secondary threshold corresponding to a 5% false-positive rate (Figure S3C), and (ii) their local orientation and period values deviate from the seed region no more than Δθ ≤ 15°, and Δ*d* ≤ 10 nm, respectively. Using this refined segmentation method, the heatmaps were subsequently converted into binary maps distinguishing the 1D network from non-1D network regions. F1 metric analysis demonstrated that the integrated 1D regularity score outperformed all other four scores, achieving the highest accuracy and sensitivity in distinguishing 1D islands from random or 2D-distributed nodes (Figure 3D,E).

To assess the sensitivity of the optimized 1D Network Classifier Module, simulated SMLM images containing 1D periodic networks were perturbed with controlled experimental noise, including lateral positional deviations of nodes, added false-positive nodes, missed node detections, background localization noise, and variation in the per-node localization number. The F1 metric analysis indicates that among the five noise types, the 1D Network Classifier Module was very robust to added false-positive nodes, background localization noise, and variation in the per-node localization number. In contrast, lateral positional deviations of nodes and missed node detections had much stronger impact and caused a more pronounced reduction in the F1 metric for identifying 1D regularity (Figure S3D).

We then applied the 1D Network Classifier Module to experimental SMLM images of hippocampal neuron dendrites immunostained for actin nodes (i.e., the N-terminus of βIII-spectrin), which have been shown to exhibit mixtures of 1D, 2D, and non-network distributions in neurons. The resulting segmentation maps showed that regions classified as a 1D network formed a subset of the broader “network” classified by the Connectivity Classifier Module, corresponding to areas with visually apparent 1D periodicity (Figure 3F–J). The distribution of measured periods across all identified 1D periodic network regions peaked at ∼188 nm (Figure 3H,K), consistent with previous reports.^4,5^ Compared to the previously developed 1D autocorrelation analysis,^20^ which requires manual selection of analyzed segments (segment length ≥2 μm to ensure reliable autocorrelation calculation) followed by projection of fluorescence signals onto the axis of the axonal or dendritic segment to calculate 1D autocorrelation amplitude as the measure for the degree of 1D periodic order, our module provides a much finer spatial resolution of 0.256 μm while simultaneously outputting period and orientation heatmaps. Notably, the 1D Network Classifier Module can identify 1D periodic subregions within neurite segments whose overall 1D autocorrelation amplitude would have classified the entire segment as a non-1D network (Figure S3E,F), demonstrating more sensitive and spatially precise detection of 1D networks compared to traditional autocorrelation analysis. Importantly, despite this higher resolution and sensitivity, our quantification correlated strongly with the autocorrelation method (r = 0.70, Figure 3L).

### 2D Network Classifier Module to Quantify the Regularity of 2D Networks

In the cellular regions classified as “network” by the Connectivity Classifier Module, we next aimed to further identify and quantify the 2D polygonal ordered network, in addition to the classification of the 1D periodic network. We reasoned that for any given node in a 2D polygonal network its geometric features relative to its immediate neighboring nodes should resemble those of its neighbors more closely in an ordered network than in a disordered network. Therefore, to quantify the regularity (i.e., the degree of order) of 2D polygonal networks, we assessed the degree of similarity among neighboring nodes within the reconstructed node-link network (Figure 4A).

**Figure 4:**
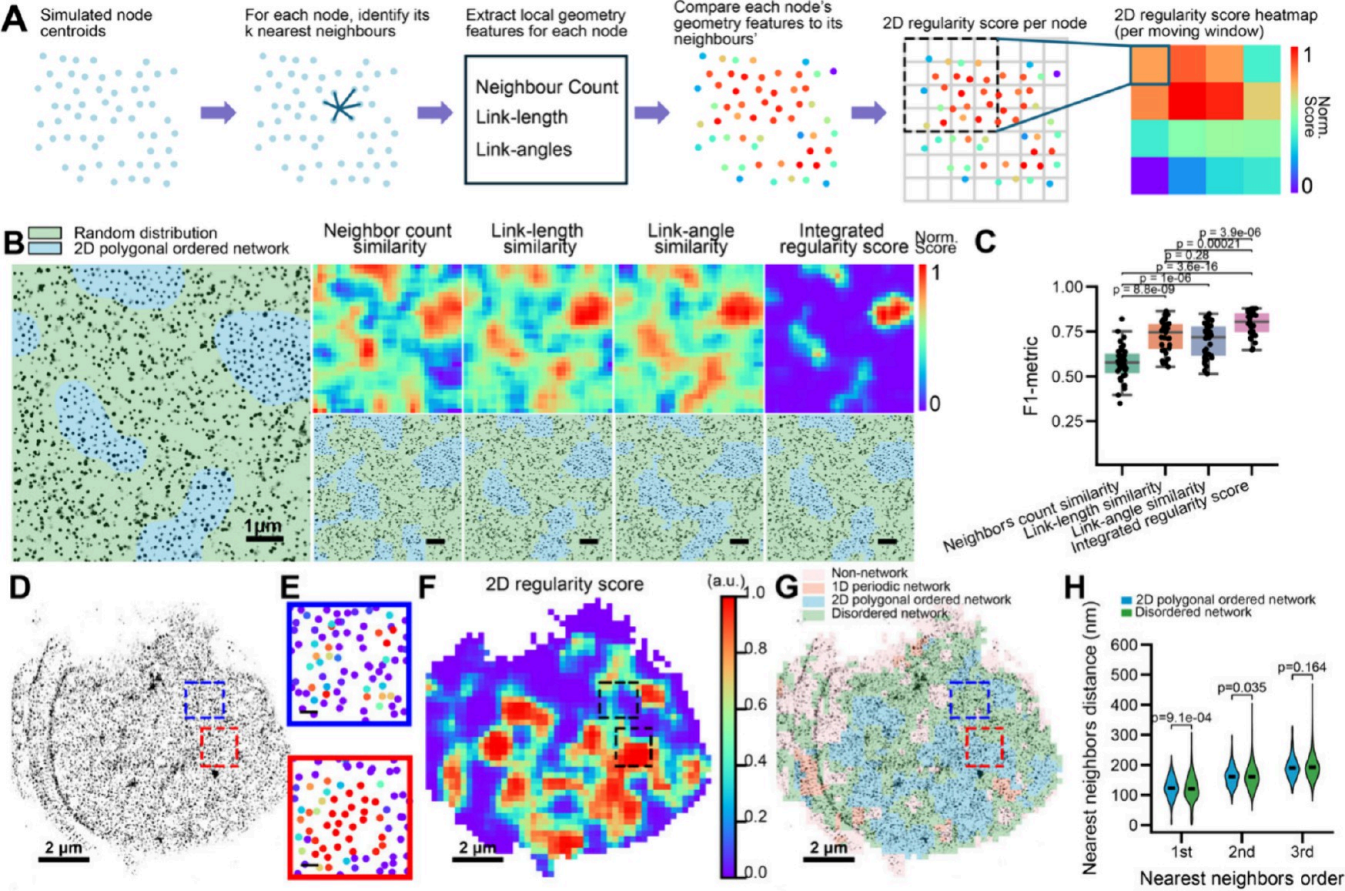
2D Network Classifier Module for quantification of 2D regularity and segmentation of 2D polygonal ordered network regions. (A) Schematic illustrating the steps of the 2D Network Classifier Module. Within the moving window (1024 nm × 1024 nm), the Connectivity Classifier Module was applied to reconstruct the node-link network. From the reconstructed network, three geometric features were calculated for each node: the number of connected neighboring nodes, the distribution of link lengths, and the distribution of link angles. The 2D regularity score for each node was defined as the inverse of the deviation of these geometric features from those of its immediate neighboring nodes, thereby quantifying the geometric self-consistency within local neighborhoods. The node-based 2D regularity scores were averaged across each moving window to generate 2D regularity score heatmaps. (B) Left: representative simulated SMLM node image containing embedded islands of nodes arranged in a 2D polygonal network, surrounded by randomly distributed nodes of equal density. Right: heatmaps (top) and segmented images (bottom) generated for the simulated SMLM image on the left, using the four candidate 2D regularity scoring methods. Scale bars: 1 μm. (C) Boxplots of F1-metrics quantifying the segmentation performance in distinguishing 2D polygonal network regions from random distributions. (D) Representative experimental SMLM image of βIII-spectrin (immunolabeled at its N-terminus). Scale bars: 2 μm. (E) Magnified views of the boxed region in (D) showing the reconstructed network with each node displaying the color-coded 2D regularity score. (F,G) 2D regularity score heatmap (F) and segmented image (G) generated for the SMLM image in (D). (H) Violin plots of the first, second, and third nearest-neighbor distances (NNDs), determined for 2D polygonal ordered network and disordered network regions. The center lines indicate the median. *p*-values were calculated using a two-sided unpaired Student’s *t*-test.

To quantify 2D polygonal network regularity, we applied the same moving-window approach described above (Figure 1C), partitioning the reconstructed node-link network from the SMLM node image into local grids, thereby enabling the assessment of geometric consistency among nodes within each window. Specifically, for each moving window, we calculated four candidate 2D regularity scores based on three geometric features calculated for each node after network reconstruction from SMLM node images using the Connectivity Classifier Module. (1) **Neighbor count similarity**: the first geometric feature is the number of neighboring nodes directly connected to the given node. The 2D regularity score is defined as the inverse of the deviation in this neighbor count between a given node and its immediate neighbors. (2) **Link-length similarity**: the second geometric feature is the distribution of link lengths connecting the given node to its immediate neighbors. The corresponding 2D regularity score is defined as the inverse of the deviation in link-length distributions between a given node and its neighboring nodes. (3) **Link-angle similarity**: the third geometric feature is the distribution of angles formed between the links connected to the given node. The 2D regularity score is defined as the inverse of the deviation in angular distributions between a given node and its immediate neighbors. and (4) **Integrated 2D regularity score**: a composite 2D regularity score that simultaneously incorporates all three geometric features above. To determine the optimal combination of geometric features for distinguishing ordered from disordered networks, we employed Linear Discriminant Analysis (LDA). Using simulated ground-truth data sets containing 2D ordered and disordered networks, we trained an LDA model to identify the linear combination of the three individual 2D regularity scores that maximized the separation between ordered and disordered network classes. For each moving window, each 2D regularity score was calculated as the average value of all nodes within the moving window, generating a heatmap that represents the local degree of 2D regularity.

To compare the performance of these four 2D regularity scores, we simulated SMLM node images containing islands of nodes with 2D polygonal distributions embedded within backgrounds of either randomly distributed or 1D periodic nodes at identical node densities (Figure 4B, Figure S4A). Each 2D regularity score was applied to convert the simulated SMLM node image into a heatmap of 2D regularity, which was then thresholded to generate a binary segmented image. Quantitative comparison using the F1 metric showed that the integrated 2D regularity score performed best in distinguishing 2D ordered networks from random node distributions (Figure 4C), whereas the link-angle similarity-based regularity score performed best in distinguishing 2D ordered networks from 1D periodic networks (Figure S4A-C). However, all four 2D regularity scores were generally less effective at distinguishing 2D networks from 1D periodic networks than from random node distributions. Based on these results, we designate the integrated 2D regularity score, applied within the moving-window framework, as the 2D Network Classifier Module, which is most effective when applied after 1D periodic regions have been identified and removed by the 1D Network Classifier Module.

To assess the sensitivity and robustness of the optimized 2D Network Classifier Module, simulated SMLM images containing 2D polygonal networks were perturbed with controlled experimental noise, including lateral positional deviations of nodes, added false-positive nodes, missed detections, background localization noise, and variation in per-node localization number, analogous to the tests performed for the Connectivity and 1D Network Classifier Modules. The F1 metric decreased progressively with increasing noise levels across all five noise types (Figure S4D). Compared with the 1D Network Classifier Module, the 2D Network Classifier Module exhibited lower robustness to added false-positive nodes, background localization noise, and variation in per-node localization number; additionally, relative to the Connectivity Classifier Module, it showed reduced robustness to added false-positive nodes.

Finally, we integrated all three modules into a complete analysis pipeline, referred to as **CLaSSiNet**, which segments an SMLM node image into four categories of cellular regions corresponding to distinct network organizational states: 1D periodic network, 2D polygonal ordered network, disordered network, and non-network. The workflow proceeds as follows: (1) apply the Connectivity Classifier Module to segment the SMLM image into network and non-network regions; (2) apply the 1D Network Classifier Module to identify 1D ordered networks within the network regions; (3) apply the 2D Network Classifier Module to the remaining network regions to identify 2D ordered networks; (4) define the residual network regions as disordered networks.

Applying CLaSSiNet to our SMLM node images of the MPS network in neurons revealed distinct subregions corresponding to these four network organizational states (Figure 4D–G). We then performed nearest-neighbor distance (NND) analysis and found that 2D polygonal-ordered network regions exhibited NND values comparable to those of disordered network regions (Figure 4H).

### Performance of Classifier Modules on SMLM Images Labeling Link-Midpoints Instead of Nodes

As immunofluorescence-based SMLM often visualizes antigens as point localizations on node-link networks, we next examined how our three classifier modules would perform when the labeled antigen is located not at the nodes but at the midpoints of links. Using the MPS network as a model system, we compared antibodies recognizing the N-terminus of βII- or βIII-spectrin, which localize to actin nodes, with antibodies recognizing the C-terminus of βII-spectrin, which localizes to the midpoints of spectrin tetramers. In a 1D periodic network, SMLM images of link-midpoints are expected to exhibit periodic patterns similar to those of SMLM node images. By contrast, in a 2D polygonal network, link-midpoint distributions differ from node distributions (Figure 5A). This raised the question of whether our three classifier modules optimized for node images would also apply to link-midpoint images and whether the two representations would yield similar classification and segmentation results.

**Figure 5:**
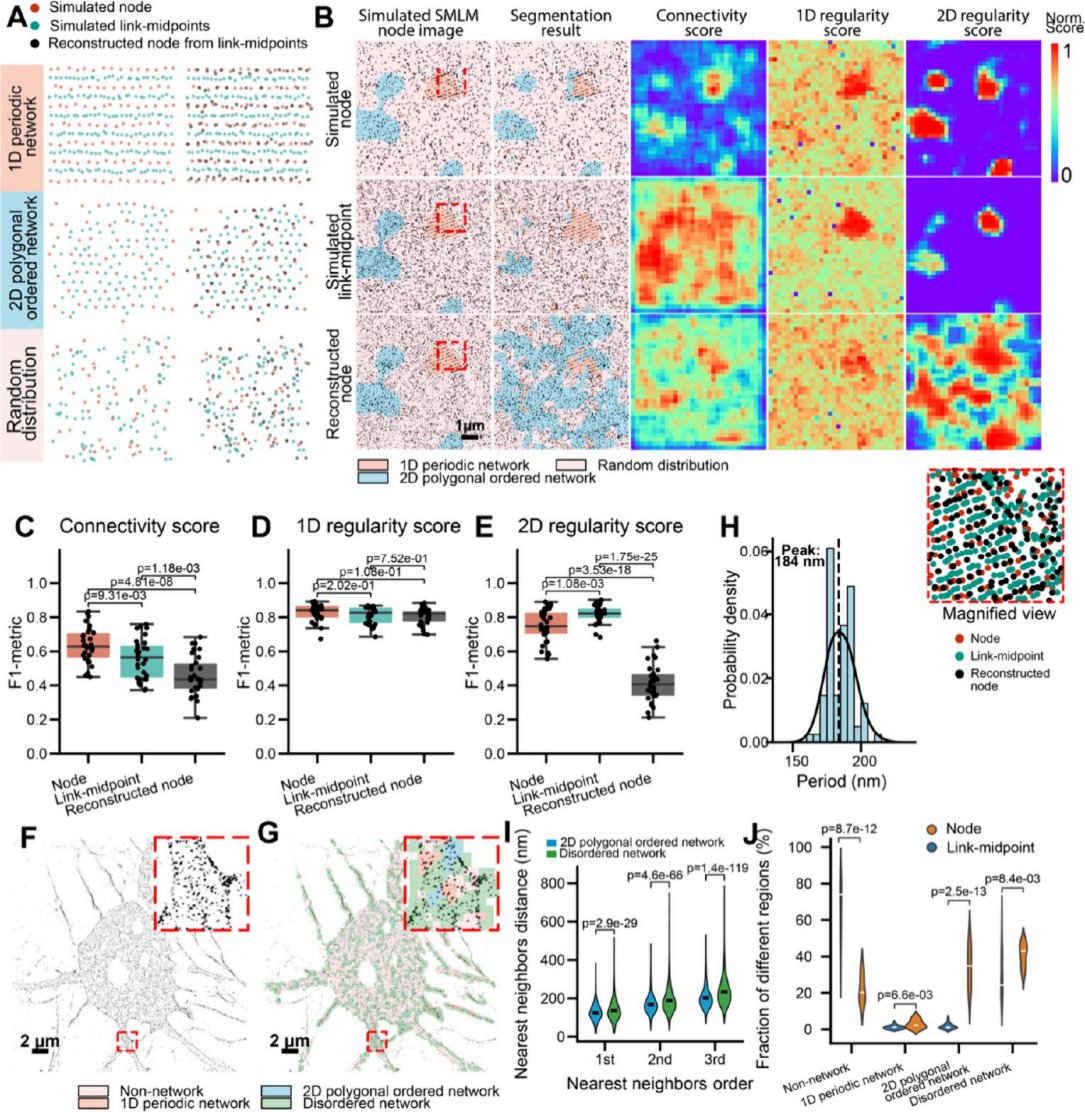
Performance of classifier modules on SMLM images labeled at link midpoints. (A) Simulated SMLM images illustrating node versus link-midpoint labeling for the same underlying networks: 1D periodic network (top row), 2D polygonal ordered network (middle row), and random distribution (bottom row). Only the centroids of molecular clusters in each SMLM image are shown. (B) Left: representative simulated SMLM image containing embedded network islands composed of molecular clusters arranged in either a 1D periodic or a 2D polygonal ordered distribution, surrounded by randomly distributed clusters. Middle and right: corresponding segmented maps (middle) as well as connectivity, 1D regularity, and 2D regularity score heatmaps (right) generated using CLaSSiNet. A magnified view of the red boxed region is shown at the bottom right corner. Scale bars: 1 μm. (C–E) Boxplots of F1 metrics quantifying segmentation performance for the three simulated SMLM image sets shown in (B): Connectivity Classifier Module (C), 1D Network Classifier Module (D), and 2D Network Classifier Module (E). (F) Representative experimental SMLM (STORM) image of βII-spectrin immunolabeled at the C-terminus (link-midpoint labeling) in a cultured neuron. (G) Segmented image corresponding to the SMLM image in (F). (H) Distribution of 1D periodicities obtained from the segmented 1D periodic regions in (F), showing a peak at ∼ 184 nm. Scale bars: 2 μm. (I) Violin plots of the first, second, and third nearest-neighbor distances (NNDs) measured from the segmented 2D polygonal ordered network and disordered network regions, with the median shown as the center line. (J) Violin plots showing the fractions of cellular area in STORM images occupied by the four distinct MPS structural states. Fractions were calculated separately for neuronal somas labeled at βII-spectrin C-termini (link-midpoints) or βIII-spectrin N-termini (nodes). Center lines indicate the median. *p*-values were calculated using a two-sided unpaired Student’s *t*-test.

To quantitatively test this, we generated three sets of simulated SMLM images containing embedded network “islands,” each composed of molecular clusters arranged in either a 1D periodic or a 2D polygonal ordered pattern, surrounded by randomly distributed clusters: (1) **Set 1**, SMLM node images with embedded islands exhibiting either 1D periodic or 2D polygonal ordered distributions; (2) **Set 2**, the corresponding link-midpoint version of Set 1, in which the same underlying networks were labeled at link midpoints instead of nodes; (3) **Set 3**, predicted SMLM node images computationally inferred from Set 2 using a Voronoi tessellation-based algorithm to estimate node locations from link-midpoint distributions (Figure S5). Set 1 served as the ground truth for evaluating the predicted node images (Set 3). We then applied the three classifier modules to all three data sets to generate connectivity, 1D regularity, and 2D regularity score heatmaps and their corresponding segmentation results (Figure 5B). Quantitative comparisons using the F1 metric revealed that directly applying the three classifier modules to SMLM link-midpoint images (Set 2) yielded better performance than predicting node positions from the same data and then analyzing the inferred node images (Set 3). Furthermore, while segmentation results from SMLM link-midpoint images (Set 2) showed comparable accuracy to those from node images (Set 1) for the 1D Network Classifier Module, they exhibited slightly reduced performance for the Connectivity Classifier Module and slightly better performance for the 2D Network Classifier Module (Figure 5C–E).

Together, these results demonstrate that our three classifier modules can robustly quantify and classify SMLM images of node-link networks regardless of whether the fluorescent label marks nodes or link midpoints and that predicting node distributions from midpoint data is less effective.

We further applied the CLaSSiNet pipeline to experimental SMLM images labeling spectrin link midpoints (i.e., C-terminus of βII-spectrin) in the somas of cultured neurons and successfully segmented the networks into 1D periodic, 2D polygonal ordered, disordered, and non-network regions (Figure 5F,G). The distribution of periods identified in the segmented 1D periodic network regions (Figure 5H) was comparable to those obtained from βIII-spectrin N-terminus-labeled (i.e., node-labeled) SMLM images (Figure 3K,H). The NND analysis of βII-spectrin C-terminus-labeled SMLM images showed that 2D polygonal-ordered network regions exhibited NND values comparable to those of disordered network regions (Figure 5I), consistent with observations from βIII-spectrin N-terminus–labeled (i.e., node-labeled) SMLM images (Figure 4H). However, when the fractions of cellular regions exhibiting different MPS organizational states were compared, βII-spectrin C-terminus labeling yielded smaller area fractions of 1D periodic, 2D polygonal-ordered, and disordered networks (Figure 5J). This likely reflects the spectrin isoform composition in neurons: βIII-spectrin is substantially more abundant than βII-spectrin in somas and dendrites of neurons, and thus βIII-spectrin labeling provides a more complete representation of the MPS network in these regions than βII-spectrin labeling.

### Orthogonal Validation and Cross-Modality Application of CLaSSiNet Using Electron Microscopy Data Set

To provide orthogonal structural validation of our network reconstruction approach and to test the applicability of CLaSSiNet beyond fluorescence-based SMLM imaging, we next analyzed an independent electron microscopy (EM) data set of the actin-spectrin membrane skeleton from erythrocyte membrane ghosts.^29^ In erythrocytes, the absence of nuclei and dense cytoplasmic organelles allows direct visualization of both actin nodes and spectrin filaments, making this system uniquely suitable for ground-truth validation of connectivity reconstruction.

We first applied the Connectivity Classifier Module to the EM image. Actin node positions and spectrin links were manually annotated based on EM-resolved networks (Figure S6AB), and Delaunay-based reconstruction was performed (Figure S6C). Predicted spectrin links were then compared directly with spectrin filaments visible in the EM image (Figure S6D). Quantitative evaluation using the F1 metric yielded a score of 0.86, indicating strong agreement between algorithm-predicted connectivity and EM-observed spectrin filaments.

Analysis of misclassifications revealed two principal scenarios. False-positive predictions primarily occurred between node pairs separated by distances within the allowable spectrin flexibility range but lacking clearly visible filaments in the EM image. These cases likely reflect either local spectrin turnover or incomplete filament preservation or visualization in the EM data set. Conversely, false negatives were most frequently associated with spectrin filaments exceeding the upper bound of the predefined link-length range, consistent with regions under elevated mechanical tension, where spectrin tetramers appear highly extended.

To further assess the sensitivity of CLaSSiNet to the allowable link-length range, we systematically evaluated the dependence of all three classifier modules on this parameter (Figure S7). We simulated SMLM node data sets containing islands of 1D periodic or 2D ordered node distributions embedded within backgrounds of randomly distributed nodes at identical densities. The three classifier modules were then applied by using varying allowable link-length ranges, and classification performance was quantified by using F1-metric analysis. These simulations indicate that the 1D Network Classifier and 2D Network Classifier Modules are largely insensitive to variations in the allowable link-length range, whereas the Connectivity Classifier Module exhibits marked sensitivity to this parameter. This result suggests that although the periodicity and geometric regularity analyses are robust to link-length constraints, biologically informed specification of the allowable link-length range remains necessary for accurate connectivity reconstruction.

We next applied the full integrated CLaSSiNet pipeline to the same EM image using the identical moving-window framework (256 × 256 nm resolution) established for SMLM data sets. Segmentation of the erythrocyte membrane skeleton revealed a predominance of ordered organizational states, with substantial fractions of both 1D periodic and 2D polygonal ordered regions and comparatively fewer disordered regions (Figure S6E). This distribution contrasts with neuronal soma SMLM data sets, where disordered and non-network regions occupy larger fractions of the cellular area. Application of the 1D Network Classifier Module to the EM data set identified periodic regions with a peak spacing of ∼162 nm (Figure S6F). Orientation, period, and 1D regularity score heatmaps generated using the 1D Network Classifier Module showed locally coherent but globally heterogeneous periodic axes (Figure S6G,H). Using the 2D Network Classifier Module, ordered regions were further distinguished from disordered regions (Figure S6J). Nearest-neighbor distance (NND) analysis showed that 2D ordered regions exhibited smaller NND values than those observed in neuronal data sets (Figure S6K).

Together, these results provide orthogonal validation of the Connectivity Classifier Module using EM-defined structural ground truth and demonstrate that CLaSSiNet can be applied across imaging modalities without modification of algorithmic parameters. This cross-modality robustness supports the generalizability of CLaSSiNet as a principled computational framework for quantitative analysis of node–link biological networks beyond fluorescence-based super-resolution microscopy.

### Application of CLaSSiNet Reveals Distinct Subcellular MPS Organizations in Epithelial and Fibroblast Cells

Having developed the integrated pipeline CLaSSiNet which incorporates three classifier modules to quantify and segment SMLM images of either nodes or link-midpoints in a node-link network, we next applied it to experimental SMLM data obtained from non-neuronal cells. CLaSSiNet classifies SMLM image regions into four distinct MPS organizational states: 1D periodic networks, 2D polygonal ordered networks, disordered networks, and non-network regions. Recent studies have revealed that the spectrin-based membrane skeleton (herein also referred to as the MPS), previously well characterized in neurons and erythrocytes using SMLM,^19,20,28^ is also present in mouse embryonic fibroblasts (MEFs).^22,30^ In MEFs, the MPS was found depleted at sites occupied by actin stress fibers, the contractile bundles of actin filaments, and myosin proteins that anchor the cell to the extracellular matrix through focal adhesions and enable cell attachment and spreading. The 1D MPS network, resembling those found in neuronal axons and dendrites, were observed between stress fibers at the adhesive surface of MEFs using expansion microscopy (ExM).^30^ However, whether similar MPS organizations exist in the epithelial cell type remains unclear.

To address this question, we first immunostained an epithelial cell line, U2OS, for two structural components of the MPS: βII-spectrin (a ubiquitous β-spectrin isoform expressed in most nonerythrocyte cell types) and F-actin.^4^ Confocal imaging revealed that βII-spectrin displayed a heterogeneous distribution at the adhesive surface, similar to that previously reported in MEFs.^30^ F-actin staining showed a mutually exclusive pattern relative to βII-spectrin, further supporting their spatial segregation. To compare MPS organization across different subcellular regions, we segmented the cell area into four distinct zones: nucleus, cell body, cell–cell junction, and cell edge (Figure 6B,C). Quantification of the average fluorescence intensities of βII-spectrin and F-actin across these subcellular zones revealed that βII-spectrin was significantly enriched at cell edges and cell–cell junctions, whereas F-actin levels remained relatively uniform across all three non-nuclear zones (Figure 6D). This observation raised the possibility that distinct MPS organizational states may occupy different proportions of the three subcellular zones. To test this, we acquired SMLM images of βII-spectrin using Stochastic Optical Reconstruction Microscopy (STORM), an SMLM imaging method,^1–3^ and applied CLaSSiNet for quantitative segmentation at a spatial resolution of 256 nm. Indeed, MPS at cell edges and cell–cell junctions contained higher fractions of 1D periodic and 2D polygonal ordered regions, whereas the cell body contained more non-network regions and was dominated by disordered networks (Figure 6E–H). We further quantified the physical properties of these organizational states: 1D periodic network regions exhibited larger periods at cell edges and cell–cell junctions compared to the cell body, while regions classified as 2D polygonal ordered had more comparable NND distributions and means across the three subcellular zones (Figure 6I,J).

**Figure 6:**
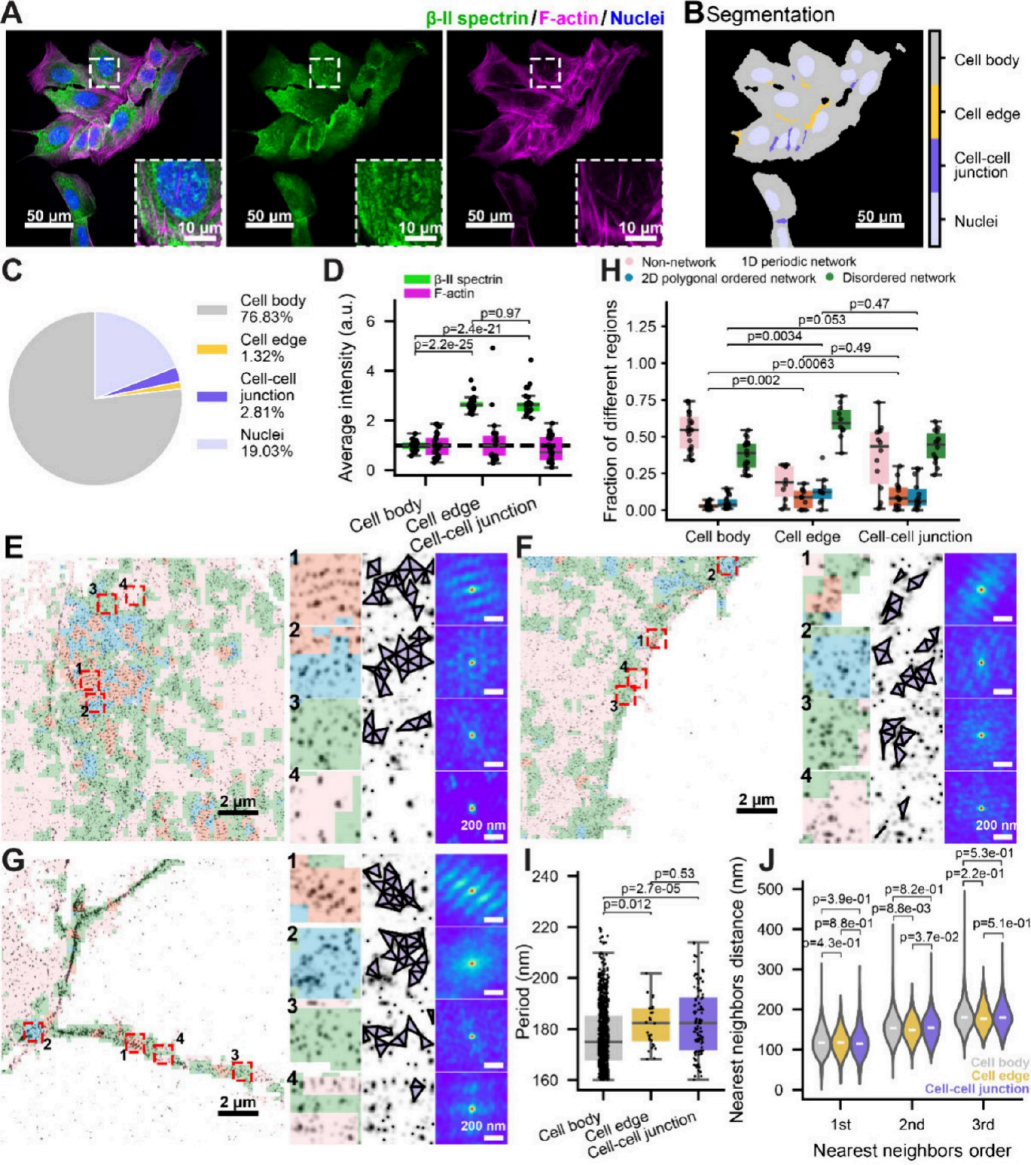
CLaSSiNet reveals distinct MPS organizations across three subcellular zones in epithelial cells. (A) Representative three-color confocal images of U2OS cells costained for nuclei (blue, Hoechst), βII-spectrin (green, βII-spectrin antibody), and F-actin (magenta, phalloidin). Scale bar: 50 μm. Inset: Magnified view of the boxed region. Scale bar: 10 μm. (B) Segmented image corresponding to (A), showing four subcellular zones: nucleus, cell body, cell edge, and cell–cell junction. Scale bar: 50 μm. (C) Quantified area fractions of the four subcellular zones. (D) Boxplots showing average fluorescence intensities of βII-spectrin and F-actin across the three non-nuclear subcellular zones (cell body, cell edge, and cell–cell junction). (E–G) Left: representative SMLM (STORM) images of βII-spectrin in three subcellular zones, cell body (E), cell edge (F), and cell–cell junction (G), overlaid with CLaSSiNet segmentation into four MPS organizational states: 1D periodic network, 2D polygonal ordered network, disordered network, and non-network regions. Scale bar: 2 μm. Middle: Magnified views of boxed regions enriched in each of the four organizational states. Right: reconstructed node-link networks and corresponding 2D autocorrelation maps confirming the expected MPS organizational patterns. Scale bar: 200 nm. (H) Boxplots showing the area fractions of the four MPS organizational states across the three subcellular zones. (I) Boxplots of 1D periodicity periods measured from 1D periodic network regions across the three subcellular zones. Boxplots show the median and interquartile range (first and third quartiles); whiskers denote the minimum and maximum values excluding outliers. (J) Violin plots comparing the first, second, and third nearest-neighbor distance (NND) distributions of 2D polygonal ordered network regions across the three subcellular zones, with the median shown as the center line. *p*-values were calculated using a two-sided unpaired Student’s *t*-test.

We next performed a parallel analysis in fibroblast 3T3 cells. Although confocal imaging showed enrichment of spectrin and F-actin at cell edges and junctions, SMLM imaging combined with our **CLaSSiNet** analysis revealed results similar to those observed in U2OS cells, except that 1D periodic and 2D polygonal ordered regions have a higher fraction only at cell edges but not at cell–cell junctions (Figure S8), likely because fibroblast cells have less defined cell–cell junctions than epithelial cells.

### Stress Fiber Proximity Influences Local MPS Organization

Recent studies indicate that the MPS and actin stress fibers are largely nonoverlapping at the cell surface (Figure 7A), leading us to hypothesize that local MPS organization, whether 1D periodic, 2D polygonal ordered, disordered network, or non-network, may depend on distance to nearby stress fibers. To test this, we performed STORM imaging to obtain SMLM images of the βII-spectrin C-terminus (located at the midpoint of a spectrin tetramer, i.e., link-midpoints) in U2OS cells, while simultaneously visualizing actin stress fibers using conventional fluorescence microscopy (Figure 7B). Applying CLaSSiNet to segment the SMLM images into four organizational states allowed us to quantify how the fraction of regions exhibiting each state varies as a function of the distance to the nearest stress fiber. We found that non-network and 1D periodic network regions were enriched near stress fibers, whereas 2D polygonal ordered network and disordered network regions were depleted near them (Figure 7E), suggesting that proximity to stress fibers promotes 1D periodic organization. We next examined whether 1D periodic regions exhibit a preferred orientation relative to stress fibers. Orientation measurements from our 1D classifier module revealed a sharp peak at ∼ 90° relative to the stress fiber orientation, indicating that the axis of MPS periodicity (i.e., orientation of the periodic pattern) is preferentially aligned perpendicular to the nearest stress fiber (Figure 7F).

**Figure 7:**
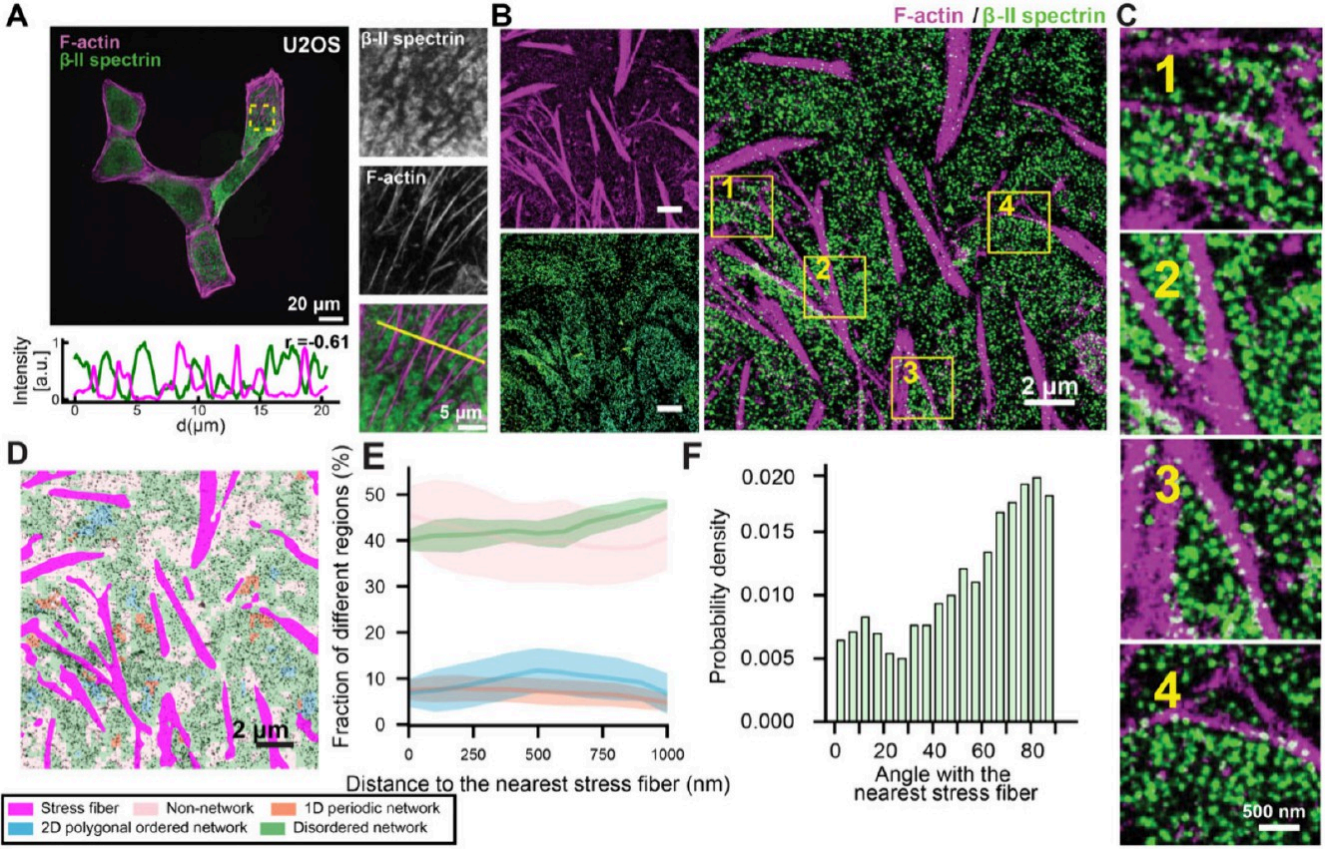
The MPS preferentially forms 1D periodic networks near actin stress fibers, with the periodicity axis oriented perpendicular to the fiber direction. (A) Top left: representative two-color confocal images of U2OS cells costained for βII-spectrin (green, βII-spectrin antibody) and F-actin (magenta, phalloidin). Scale bar: 20 μm. Right: magnified view of the boxed region. Bottom left: Fluorescence intensity profiles of βII-spectrin (green) and F-actin (magenta) along the yellow line showing anticorrelation (Pearson correlation coefficient *r* = −0.61). Scale bar: 20 μm. (B) SMLM (STORM) image of βII-spectrin (green) in a U2OS cell, overlaid with a confocal image of F-actin (magenta) from the same field of view. Scale bars: 2 μm. (C) Magnified views of the four boxed regions shown in (B). Scale bars: 500 nm. (D) CLaSSiNet segmentation of the same cell shown in (B), classifying βII-spectrin organization into four MPS structural states: 1D periodic network, 2D ordered network, disordered network, and non-network regions. Scale bars: 2 μm. (E) Cell area fractions in the SMLM images exhibiting the four different MPS structural states, plotted as a function of their distance to the nearest actin stress fibers. (F) Distribution of the relative orientation angles between actin stress fibers and the 1D periodicity axis of adjacent MPS regions, revealing preferential perpendicular alignment.

### Comparative Analysis Reveals Cell-Type-Specific MPS Organizational Signatures

Finally, we systematically compared the fractions of the four MPS organizational states across neurons, U2OS, and 3T3 cells (Figure 8A–C). Neuronal neurites exhibited the highest fraction of 1D periodic network regions, followed by U2OS cells, 3T3 cells, and neuronal somas (Figure 8D). In contrast, neuronal somas contained the largest fraction of 2D polygonal ordered regions, followed by U2OS cells, neuronal neurites, and 3T3 cells (Figure 8E). Disordered network regions showed relatively similar fractions across neuronal somas, neurites, U2OS, and 3T3 cells (Figure 8F). The average 1D period of the periodic network also varied by cell type: neuronal neurites (∼186 nm) > neuronal somas (∼183 nm) > 3T3 cells (∼182 nm) > U2OS cells (∼179 nm) (Figure 8G). These compositional and geometric differences were further reflected in the overall 1D and 2D regularity score distributions for each cell type (Figure S9).

**Figure 8:**
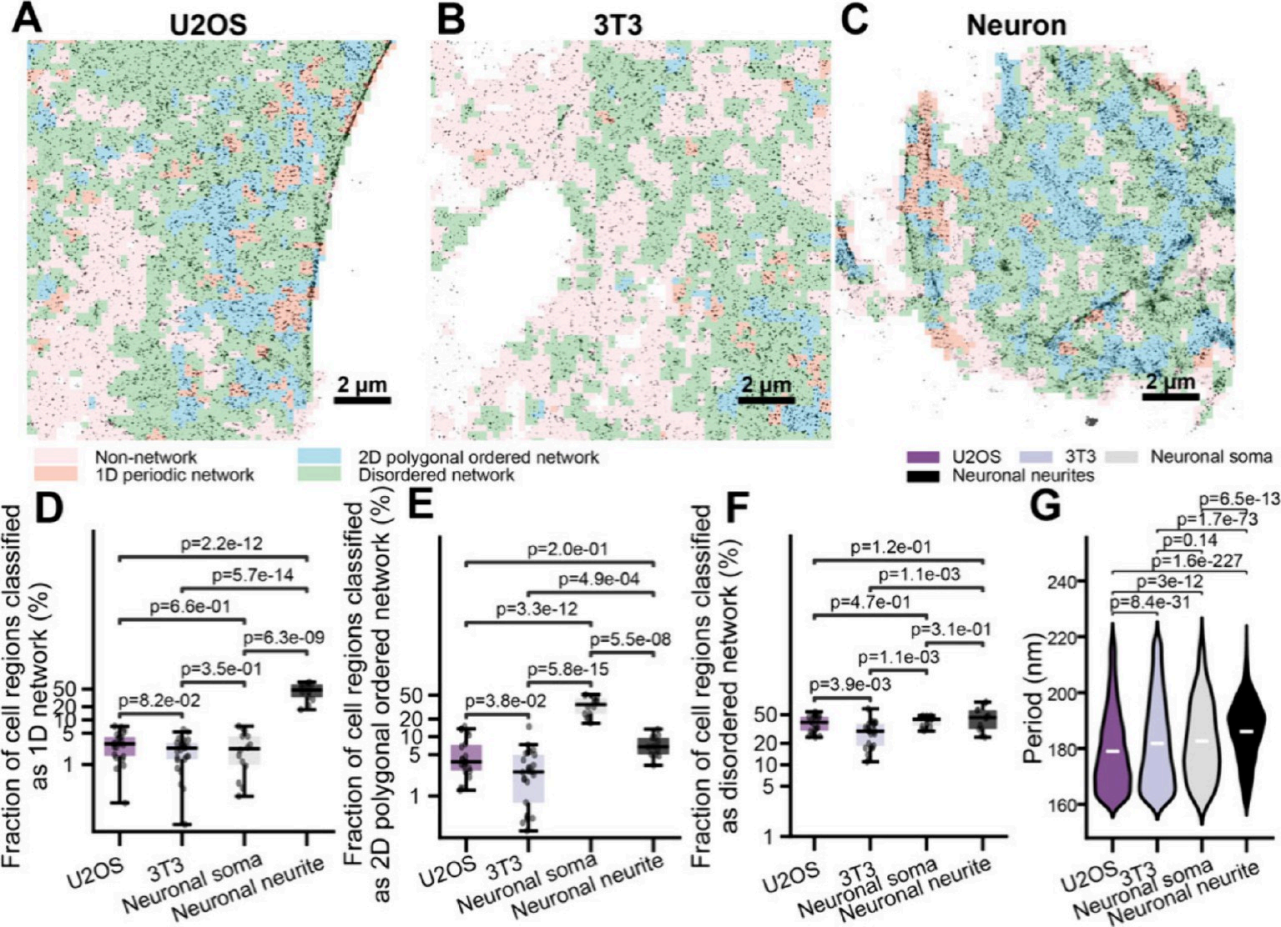
Comparative analysis reveals cell-type-specific MPS architectural signatures. (A-C) Representative SMLM (STORM) images of βII-spectrin in a cultured hippocampal neuron (A), a U2OS cell (B), and a 3T3 cell (C), overlaid with CLaSSiNet segmentation into four MPS organizational states: 1D periodic network, 2D polygonal ordered network, disordered network, and non-network regions. Scale bars: 2 μm. (D,E) Boxplots showing the cell area fractions exhibiting 1D periodic networks (D) or 2D polygonal ordered networks (E) across neuronal somas, neuronal neurites, U2OS cells, and 3T3 cells. (F) Boxplots of 1D periodicity periods measured from 1D periodic network regions across neuronal somas, neuronal neurites, U2OS cells, and 3T3 cells. Boxplots show the median and interquartile range (first and third quartiles); whiskers denote the minimum and maximum values excluding outliers. (G) Violin plots comparing the first, second, and third nearest-neighbor distance (NND) distributions of 2D polygonal ordered network regions across neuronal somas, neuronal neurites, U2OS cells, and 3T3 cells, with the median shown as the center line. *p*-values were calculated using a two-sided unpaired Student’s *t*-test.

## Discussion

Computational analysis of cytoskeletal network organization in SMLM has largely evolved around methods developed after the discovery of the MPS structures (Table S1). Early quantification approaches, including 1D autocorrelation analysis^28^ and sinusoidal fitting,^31^ were designed to measure periodic spacing along neurites and therefore detect only 1D periodic order. 2D autocorrelation analysis^20^ extended this framework to assess planar regularity but did not reconstruct network topology. Separate range-based network reconstruction strategies^20^ enabled identification of connected regions; however, these approaches focused on connectivity alone and did not quantify geometric order. More recent tools such as Gollum,^32^ SReD,^33^ and Napari-WaveBreaker^34^ improved automation and reduced bias in region-of-interest selection for 1D MPS periodicity analysis. Gollum requires user-defined period input and derives 1D periodicity orientation from overall neurite morphology rather than from local network topology, whereas SReD reports a single globally averaged periodicity value for the entire field of view and similarly infers periodicity orientation from neurite morphology instead of local structural features. Although SReD and Gollum generate spatial heatmaps of periodic strength, these tools remain specialized for detecting a single regular state—1D periodic network—against a nonregular background. None of these existing approaches integrates connectivity reconstruction, local 1D periodicity detection, and 2D geometric classification within a unified analytical framework, nor do they explicitly distinguish multiple ordered and disordered network states within the same data set.

CLaSSiNet addresses this gap by introducing a modular, multistate classification strategy that combines Delaunay-based connectivity reconstruction with local Fourier-based 1D periodicity detection and quantitative 2D geometric similarity analysis. In contrast to prior range-based reconstruction methods, Delaunay triangulation preserves local topology while minimizing arbitrary linking artifacts, and network discrimination incorporates higher-order features, such as triangle area sum. Unlike Gollum and SReD, CLaSSiNet automatically extracts the local period and orientation at 256 nm classification resolution directly from the network topology, enabling detection of heterogeneous periodic alignment independent of global neurite geometry and without requiring user-defined period inputs. For 2D organization, CLaSSiNet uniquely evaluates neighbor-count similarity, link-length similarity, and link-angle similarity to distinguish polygonal ordered networks from disordered but connected structures with comparable density. By integrating these three classifier modules, CLaSSiNet enables simultaneous discrimination of four structural states—1D periodic, 2D polygonal ordered, disordered, and non-network—thereby advancing super-resolution network analysis from single-feature detection toward comprehensive structural state classification.

Another significant advance of CLaSSiNet is its ability to analyze both link-midpoint (e.g., β-spectrin C-termini) and node (e.g., actin filaments or β-spectrin N-termini) labeling. Our analyses revealed that link-midpoint labeling is sufficient to capture connectivity, 1D periodicity, and 2D regularity. These findings highlight the generalizability of our framework across different labeling strategies while also cautioning that reconstructing node distributions from midpoint data introduces additional errors and performs worse than directly analyzing the link-midpoint distributions.

Applying CLaSSiNet to experimental data sets uncovered several new insights into the organization of the membrane-associated periodic skeleton (MPS). In epithelial (U2OS) and fibroblast (3T3) cells, we found that distinct MPS organizational states occupy different subcellular zones, with 1D periodic and 2D ordered regions enriched at cell edges and junctions, while non-network regions dominate cell bodies. Segmented 1D periodic network regions also exhibited larger periods at cell edges and cell–cell junctions compared with the cell body. As previous studies using biophysical techniques and fluorescent biosensors have shown that membrane tension is higher at cell edges^35–37^ and cell–cell junctions,^38,39^ it raised the possibility that local mechanical tension governs MPS architectural state with highly ordered 1D periodic and 2D ordered MPS structures preferentially forming under high membrane tensions.

We further discover a mechanistic insight that the proximity to actin stress fibers influences MPS organization. Specifically, the 1D periodic network was more frequently found adjacent to stress fibers, and the periodic axis was preferentially oriented perpendicular to fiber orientation. These results suggest a functional interplay between spectrin- and actin-based systems, where the MPS may provide complementary mechanical support and membrane organization in regions flanking contractile actin bundles. Such spatial coordination is likely influenced by local membrane tension, which is known to be elevated near stress fibers.^40,41^

Finally, cross–cell-type comparisons revealed distinct MPS organizational signatures across neuronal somas, neurites, U2OS cells, and 3T3 fibroblasts. Neuronal neurites contained the highest fraction of 1D periodic network regions with the longest average periodicity, whereas neuronal somas exhibited the largest fraction of 2D polygonal ordered networks. U2OS and 3T3 cells displayed intermediate compositions, with 1D periodic fractions lower than those in neurites and 2D ordered fractions lower than those in neuronal somas. These compositional and geometric differences highlight the adaptability of the spectrin-based MPS, which can assemble into cell-type–specific architectures suited to the distinct structural and functional demands of each cellular context.

Despite its robustness across multiple perturbation conditions and imaging modalities, CLaSSiNet has several limitations that users should consider. First, each classifier module exhibits distinct sensitivities to experimental noise: the Connectivity Classifier Module is most affected by substantial lateral positional deviations of nodes, missed node detections, and background localization noise, which can disrupt network reconstruction and reduce F1 metrics, whereas it remains relatively robust to added false-positive nodes and variation in per-node localization number; the 1D Network Classifier Module is particularly sensitive to lateral positional deviations of nodes and missed node detections that obscure periodicity but is largely resilient to added false-positive nodes, background localization noise, and variation in per-node localization number; the 2D Network Classifier Module shows lower robustness than the other modules to added false-positive nodes, background localization noise, and variation in per-node localization number, making accurate 2D regularity classification more challenging under these conditions. Second, connectivity reconstruction requires a biologically informed specification of the allowable link-length range; overly restrictive ranges may lead to false negatives (e.g., highly extended links under tension), whereas overly broad ranges can introduce spurious connections. Additionally, while the Connectivity Classifier Module can still quantify network-forming tendency via the connectivity score heatmap when analyzing link-midpoint-labeled data sets, the reconstructed network itself is inaccurate because the algorithm incorrectly assumes link midpoints correspond to nodes. Third, differences in labeling density or isoform abundance (e.g., βII- vs βIII-spectrin) can influence absolute measurements of the network fraction, connectivity, and classification accuracy. Finally, CLaSSiNet is optimized for networks exhibiting periodic or polygonal organization; highly heterogeneous, multiscale, or non-network spatial arrangements may fall outside the framework’s intended scope. Future extensions incorporating adaptive window sizing, probabilistic modeling, or automated parameter optimization could further improve the performance under challenging experimental conditions.

Together, these findings establish CLaSSiNet as a versatile and quantitative framework for the multidimensional analysis of nanoscale networks. Beyond the membrane skeleton, this modular pipeline is readily adaptable to other supramolecular assemblies including junctional complexes, organellar scaffolds, and diverse cytoskeletal networks imaged via SMLM or emergent super-resolution modalities. By enabling the systematic mapping of nanoscale organization across distinct cell types and subcellular domains, CLaSSiNet provides a rigorous foundation for uncovering the fundamental design rules of cellular networks and their remodeling under mechanical stress or disease states. Ultimately, by bridging computational topology with high-resolution cell biology, CLaSSiNet offers a powerful strategy for dissecting how nanoscale spatial patterning dictates macroscopic cellular mechanics, signaling, and homeostatic function.

## Conclusion

In summary, we have developed CLaSSiNet, a modular computational framework that enables the robust quantification and classification of nanoscale molecular networks from super-resolution microscopy data. By integration of connectivity, 1D periodicity, and 2D regularity classifiers, CLaSSiNet accurately distinguishes between distinct topological organizations and remains resilient to experimental noise and variations in labeling chemistry. Using actin–spectrin MPS as a model supramolecular system, we demonstrated that this approach reveals cell-type-specific and subcellular variations in lattice architecture. Furthermore, we uncovered a mechanical coordination principle between the MPS and actin stress fibers, wherein 1D periodic networks are preferentially templated near contractile bundles and exhibit an orthogonal alignment relative to the fiber axis. These findings underscore the architectural plasticity of spectrin-based scaffolds in response to local mechanical environments and establish CLaSSiNet as a powerful tool for the quantitative mapping of the nanoscale molecular order. More broadly, this framework establishes a rigorous foundation for elucidating the fundamental design principles governing the hierarchical assembly and spatial organization of complex supramolecular networks across diverse biological and bioinspired interfaces.

## Experimental Section

### Antibodies

The following primary antibodies were used in this study: mouse anti-βII spectrin antibody 1:200 dilution for IF (Santa Cruz, sc-515592), mouse anti-βII spectrin antibody 1:200 dilution for IF (BD Biosciences, 612563), goat anti-βIII spectrin antibody 1:100 dilution for IF (Santa Cruz, sc-9660), and mouse anti-βIII spectrin antibody 1:100 dilution for IF (Santa Cruz, sc-515737).

The following secondary antibodies were used in this study: Alexa Fluor-647-conjugated donkey antimouse IgG antibody 1:800 for IF (Jackson ImmunoResearch, 715-605-151), Alexa Fluor-647-conjugated donkey antigoat IgG antibody 1:800 for IF (Jackson ImmunoResearch, 705-605-147), CF583R-conjugated secondary antibodies were made by conjugating the unlabeled secondary antibodies with CF583R succinimidyl ester (Biotium, 96084) to achieve a labeling efficiency of ∼2 dyes per antibody, as previously described.

### Cell Culture

U2OS and 3T3 cells were grown in DMEM (Gibco; 11966025) supplemented with 2 mM l-glutamine (Gibco; 25030081), 10% fetal bovine serum (Fisher Scientific; SH3008802HI), and 1% penicillin/streptomycin (Gibco; 15140122). Cultures were maintained in a humidified incubator at 37 °C in 5% CO_2_. The cells were plated on precleaned 18 mm coverslips at 10–20% confluency. After 24–48 h, the cells were fixed and permeabilized for immunostaining.

All animal procedures were performed in compliance with the National Institutes of Health Guide for the Care and Use of Laboratory Animals and were approved by the Pennsylvania State University IACUC. Primary cultures of hippocampal neurons were prepared as previously described.^16,20,23^ Briefly, timed-pregnant CD-1 IGS mice (Charles River Laboratories, Wilmington, MA; 022) were euthanized, and the hippocampi were isolated from E18 mouse embryos. The dissected tissues were enzymatically dissociated with 0.25% trypsin-EDTA (1×) (Sigma, T4549) at 37 °C for 15 min. Following digestion, the hippocampal tissues were washed three times with Hanks’ Balanced Salt Solution (HBSS) (Thermo Fisher Scientific, 14175095), and then transferred to NbActiv1 culture medium (Transnetyx Tissue, NB1), a premixed formulation comprising Neurobasal, B27, and Glutamax, with the addition of 100 μg/mL Primocin (InvivoGen, ant-pm-2). The tissues were gently triturated in the culture medium until a single-cell suspension was achieved, ensuring that no tissue clumps remained. Dissociated cells were then counted and plated onto poly-d-lysine-coated 18 mm coverslips (Neuvitro, GG-18-1.5-PDL). Cultures were maintained in a humidified incubator at 37 °C with 5% CO_2_. Half of the medium volume was replaced every 5 days to maintain optimal culture conditions. Neurons were fixed between 21 and 28 days in vitro for the subsequent experiments described in this study.

### Fixation and Fluorescence Labeling of Cells

Cells were fixed with 4% (w/v) PFA and 4% sucrose in PBS for 15–30 min. The fixed cells were blocked with a blocking buffer comprising 3% (w/v) BSA (Fisher Bioreagents; 9048468), 0.5% (v/v) Triton X-100 (Sigma-Aldrich; X100) in PBS (Thermo Scientific; J61196.AP) for 20 min and then incubated with the primary antibodies in the blocking buffer overnight at 4 °C, followed by three washes with PBS. The cells were further incubated with the corresponding dye-conjugated secondary antibodies for 60 min at room temperature (RT), washed thoroughly with PBS four times at RT, and then stored at 4 °C. To label actin stress fibers, cells were incubated with CF583R- or AF647-conjugated phalloidin (500 nM in PBS; Biotium, 00064; Thermo Fisher, A22287) either overnight at 4 °C or for 1–4 h at room temperature, followed by three washes with PBS.

### STORM Imaging

The STORM setup was built on a Nikon Eclipse-Ti2 inverted microscope, equipped with a 100× (N.A. 1.45) Plan Apo oil-immersion objective (Olympus) and an EM-CCD camera (Andor iXon Life 897). 405 nm (Coherent, OBIS 405 nm LX, 1265577; 140 mW), 488 nm (Coherent, Sapphire 488–500 LPX CDRH, 1416094; 300 mW), 560 nm (MPB Communications Inc., 2RU-VFL-P-1500-560-B1R; 1500 mW) and 642 nm (MPB Communications Inc., 2RU-VFL-P-2000-642-B1R; 2000 mW) lasers were introduced through the back port of the microscope. The laser beams were shifted toward the edge of the objective to achieve near-total internal reflection illumination, ensuring the selective excitation of fluorophores within a few micrometers of the coverslip surface. For single-color STORM imaging, AF647 was imaged. A cylindrical lens was inserted into the detection path to introduce ellipticity into the point spread function, allowing determination of the *z*-positions of single molecules from the ellipticity of their images. Samples were imaged in an oxygen scavenging imaging buffer containing 100 mM Tris-HCl (pH 7.5), 100 mM cysteamine (Sigma-Aldrich, M9768-50G), 5% (w/v) glucose (Sigma, G8270-100G), 0.8 mg/mL glucose oxidase (Sigma-Aldrich, G2133-10KU), and 40 μg/mL catalase (Sigma-Aldrich, C100-50MG). During imaging, continuous illumination of 642 or 560 nm laser (∼2 kW/cm^2^) was used to excite AF647 or CF583R molecules, respectively, switching them into the dark state. Continuous illumination of the 405 nm laser (0–1 W/cm^2^) was used to reactivate the fluorophores to the emitting state, and the illumination power was controlled so that at any given time only a small, optically resolvable fraction of the fluorophores in the field of view were in the emitting state. A typical single-color STORM image was reconstructed from ∼30,000 image frames acquired at a frame rate of 110 Hz. Super-resolution images were reconstructed from the molecular coordinates by depicting each location as a 2D Gaussian peak.

### STORM Image Preprocessing

Raw SMLM data from STORM imaging were processed to generate localization coordinates using Insight3 as previously described.^16,20,23^ The rendered STORM images were generated by binning the SMLM localization data into a 2D histogram with a pixel size (bin size) of 16 nm. This value was chosen to be on the order of the estimated localization precision (∼10–20 nm) and to comfortably satisfy the Nyquist sampling theorem for the finest periodic features of interest (∼95 nm), which requires a pixel size smaller than approximately 47.5 nm. The resulting 2D histogram was then used to produce the final rendered STORM image.

To identify individual molecular clusters from SMLM localization data, we implemented a two-pass, coarse-to-fine strategy balancing computational efficiency and precision. In the first pass, Laplacian of Gaussian (LoG) blob detection (scikit-image blob_log) was applied to contrast-enhanced rendered STORM images (16 nm/pixel) to rapidly identify candidate regions of interest likely containing molecular clusters. The algorithm searched across multiple scales using 20 logarithmically spaced levels with intensity thresholds of 0.1. In the second pass, for each candidate ROI, we extracted the corresponding raw localization coordinates (<20 nm precision) from the original data set within a 50 nm search radius. HDBSCAN (Hierarchical Density-Based Spatial Clustering, min_cluster_size = 10, min_samples = 5) was then applied to this subset to accurately define final cluster membership, automatically discarding outlier localizations and determining precise cluster centers directly from the nanoscale point cloud. This hybrid approach leverages the speed of image-based detection for large fields of view while retaining the nanometer-scale accuracy of point-cloud clustering, avoiding information loss from image rendering.

### SMLM Image Simulations

To evaluate CLaSSiNet under realistic imaging conditions, we generated synthetic single-molecule localization microscopy (SMLM) data sets that emulate network-like cellular structures at the level of individual molecular detection events. Network elements were represented as localization clusters, where each cluster corresponds to either a node (e.g., a filament junction) or a link-midpoint (e.g., the midpoint of a spectrin tetramer connecting adjacent nodes). Each cluster consisted of multiple simulated localizations distributed within a small radius around its centroid, capturing the spatial spread expected from the experimental SMLM imaging.

To ensure realism, key simulation parameters were empirically derived from representative experimental data sets (Figure S1). First, we quantified fluorophore blinking statistics by fitting Log-Normal distributions to the experimental measurements of (1) the number of localization events per molecular/localization cluster (Figure S1A) and (2) the localization precision of single emitters (Figure S1B). These fitted distributions were sampled during simulation to assign the number of localizations and per-localization positional uncertainty for each cluster, thereby reproducing realistic SMLM variability.

Simulated networks were initially constructed as perfect ordered structures: parallel lines with equal spacing (e.g., 190 nm for 1D periodic network^20^ and hexagonal lattices for 2D polygonal ordered networks). We then introduced experimentally motivated perturbations, including a missed-detection rate, a false-positive localization rate, and lateral positional deviations of cluster centroids. These perturbations were systematically tuned so that the simulated data sets reproduced the experimentally observed distributions of nearest-neighbor distances (NNDs) for each organizational state (Figure S1C,D). Additionally, the average spacing between adjacent cluster centroids perpendicular to the 1D periodicity axis was measured from experimental data (Figure S1E,F) and used to set the geometric scale of the simulated 1D periodic network. Finally, non-network (random) organization was produced using a Completely Spatially Random (CSR) Poisson process, in which localization clusters were placed independently and uniformly within a defined area to match empirically measured localization cluster densities. All simulations used this set of empirically constrained parameters and geometry rules, and thus provide a controlled yet biologically faithful basis for method validation and benchmarking.

### Generation of Composite Simulated SMLM Images

To assess segmentation performance at interfaces between distinct network organizations, we generated composite SMLM images by combining localization clusters with different structural patterns across irregular boundaries. These boundaries were defined using a multistep procedure: random noise generation, Gaussian blurring to establish the spatial scale of features, thresholding, and morphological refinement to create smooth, nonlinear masks. Each mask delineated two complementary regions within the image, which were then populated with localization clusters representing different organizational states (e.g., 1D periodic network versus random distribution, or 2D polygonal ordered network versus random distribution) at matched cluster densities. This approach produced composite data sets with known ground-truth spatial domains, enabling quantitative benchmarking of segmentation accuracy across domain interfaces.

### Reconstruction of Node Positions Based on Link-Midpoint Positions

To evaluate how node positions (e.g., labeled at the β-spectrin N-terminus for the MPS network) in a biological node-link network can be inferred from SMLM images of link midpoints (e.g., labeled at the β-spectrin C-terminus for the MPS network), we developed and tested an algorithm that computationally reconstructs node positions using only the centroid coordinates of link-midpoint localization clusters. The algorithm proceeds in several steps (Figure S5). First, initial candidate node positions were identified as vertices of a Voronoi tessellation constructed from the link-midpoint centroids because these vertices, similar to biological nodes, are equidistant from neighboring link-midpoint clusters. Candidate nodes were then geometrically filtered, retaining only those whose distances to the surrounding link-midpoint centroids fell within a physically plausible range. If the internodal distance was smaller than the effective image resolution, then the corresponding candidate nodes were consolidated into a single representative node. Finally, an iterative network-assembly step refined the predicted node set: starting from the highest-confidence nodes (i.e., those connected to multiple link midpoints), the algorithm enforced the constraint that each link midpoint connects exactly two nodes. Missing N-terminal partners were computationally added where geometrically feasible to complete N–C–N spectrin tetramer structures. This workflow produced a final predicted map of N-terminal node positions, which was subsequently used for downstream network reconstruction and validation across 1D periodic, 2D polygonal ordered, and disordered network patterns.

### Cellular Area Determination in STORM Images

Cell boundaries were identified from rendered STORM images using fluorescence-intensity–based segmentation. The rendered SMLM image was first divided into a grid with a pixel pitch of 256 × 256 nm. A sliding analysis window (4 × 4 pixels; step = 1 pixel) was then applied to scan the entire image, computing the total fluorescence intensity within each window to generate an intensity heatmap. Pixels with summed intensities below a defined threshold (<10 arbitrary units) were excluded from subsequent classifier analyses to eliminate regions dominated by background signal. Contour detection was then applied to the retained regions, and only continuous areas exceeding a minimal size (∼5000 nm^2^) were preserved to further remove noise and spurious detections. This procedure ensured that downstream analyses were restricted to high-confidence cellular areas. For neurite–soma segmentation, morphological opening was applied to the cell masks to selectively remove thin neurites while preserving the larger soma region.

### Connectivity Classifier Module

To classify local regions as network or non-network from SMLM data, we developed a connectivity classifier module that reconstructs node–link organization from localization cluster centroids and quantifies connectivity within spatial windows. Two reconstruction strategies were implemented: range-based and Delaunay-based. In the range-based method, centroid pairs whose separations fell within biologically constrained distances (160–220 nm for SMLM node images, 40–220 nm for SMLM link-midpoint images, and 120–220 nm for EM images) were connected as putative spectrin links; intersecting links were resolved by randomly removing one. In the Delaunay-based method, a Delaunay triangulation was generated from the centroids, and links (i.e., triangle edges) outside the same distance windows were removed except when they were the sole out-of-range link in a triangle whose remaining two links were in range, preserving characteristic triangular motifs.

Connectivity was quantified using three metrics normalized per unit area: (1) link count, (2) triangle count, and (3) triangle area sum. These metrics capture pairwise connectivity, closed-network motifs, and geometric extent, producing six reconstruction–quantification pipelines (2 reconstruction methods × 3 metrics), among which the Delaunay-based method combined with triangle area sum is designated as the optimized connectivity score.

To resolve spatial heterogeneity, a sliding analysis window (1024 × 1024 nm; step = 256 nm) was applied to the rendered SMLM or EM image: each window was independently reconstructed and quantified, and the resulting values were mapped back to generate a connectivity score heatmap across the cell. To identify genuine network domains rather than density-driven artifacts, connectivity values in each window were assessed against density-matched null models. For every window, 100 simulated random centroid distributions were generated at the same centroid density and a connectivity threshold was defined as the 95th percentile of the simulated distribution. Sliding windows with connectivity scores exceeding this density-dependent threshold were classified as significantly connected (5% false-positive rate; *p* < 0.05), enabling robust detection of MPS network regions.

### 1D Network Classifier Module

To identify 1D periodic network regions, each SMLM or EM image was divided into grids using a moving window (1024 × 1024 nm; step = 256 nm). Within each window, a 1D periodic network was detected using Fourier-based spectral analysis. Each window was preprocessed by normalization, multiplication with a 2D Hann window to minimize spectral leakage, and zero-padding, followed by a 2D Fast Fourier Transform (FFT) to the frequency domain. The dominant frequency peaks were isolated using an annular mask corresponding to real-space periods of 160–220 nm, and for each window, the orientation (θ) and period (*d*) were extracted using the inverse FFT which generated a grating image representing the best-fitting orientation and period of the candidate 1D network.

Five candidate 1D regularity scores were computed for each window to quantify the degree of 1D periodic order: (1) FFT peak amplitude (*A*_*FFT*_), the amplitude of the two dominant frequency peaks in the 2D frequency domain image; (2) 2D Pearson correlation (*r*_*global*_), the Pearson correlation coefficient between the raw SMLM image and the invFFT-generated grating image; (3) 1D projected Pearson correlation (*r*_*proj*_), the Pearson correlation coefficient between 1D signal profiles obtained by projecting both images in (2) along the grating axis; (4) localized 2D Pearson correlation (*r*_*local*_), similar to (2), but the correlation is calculated between the raw SMLM image and a cropped, elongated grating image to account for cases where the 1D periodic network occupies only a subregion within the moving window. To robustly capture 1D network subregions that do not span the entire moving window, the dimensions of this template are tunable. Specifically, the template width is empirically set to 6 pixels to encapsulate the characteristic spatial span of the localized molecular clusters (preventing artifacts from single-point noise), while the template length is set to 3 local periods to ensure sufficient spatial repetition for reliable periodicity detection.; (5) Integrated 1D regularity score (*S*_*integrated*_), a composite metric that mathematically integrates measures (2) and (4) using a probabilistic “Logical OR” operation to account for both large 1D network islands spanning the full window and small local 1D regions. The integration is defined as

$$S_{integrated}=r_{global}+r_{local}-\left(r_{global}\times r_{local}\right)$$

Among the five scores, the integrated 1D regularity score is designated as the optimized 1D regularity score.

For each moving window, the optimized 1D regularity score was computed, and an initial threshold (>0.6) corresponding to a 1% false-positive rate (*p* < 0.01) was applied to identify high-confidence “seed” pixels as candidate 1D periodic network regions. These seed regions were then expanded via a spatially coherent region-growing algorithm: neighboring pixels were annexed iteratively if their score exceeded a secondary threshold (>0.45) corresponding to a 5% false-positive rate (*p* < 0.05) and their orientation and period values were consistent with the seed (Δθ < 15° and Δ*d* ≤ 10 nm). Postfiltering removed segmented regions below a predefined size, unifying fragmented domains, and eliminating spurious pixels. Finally, the refined segmentation produced binary maps distinguishing the 1D periodic network from non-1D regions.

### 2D Network Classifier Module

To quantify 2D polygonal network regularity, we applied the same moving-window approach (1024 × 1024 nm; step = 256 nm), partitioning the SMLM or EM images into local grids. For each window, we calculated four candidate 2D regularity scores based on three geometric features derived from the reconstructed network using the Connectivity Classifier Module. (1) Neighbor count similarity—the number of nodes directly connected to a given node. The 2D regularity score is defined as the inverse of the deviation in neighbor count between a node and its immediate neighbors. (2) Link-length similarity—the distribution of lengths of links connecting a node to its neighbors. The score is the inverse of the deviation in link-length distributions between a node and its neighbors. (3) Link-angle similarity—the distribution of angles formed between links connected to a node. The score is the inverse of the deviation in angular distributions between a node and its neighbors. (4) Integrated 2D regularity score—a composite metric combining all three geometric features to capture overall local network regularity. To optimize the combination of these features for distinguishing ordered from disordered networks, we trained a Linear Discriminant Analysis (LDA) model using simulated ground-truth data sets containing 2D ordered and disordered networks. For each moving window, the 2D regularity score of the window was calculated as the average score of all nodes contained within it, generating a heatmap representing the local degree of 2D regularity. This approach enables robust identification and mapping of polygonally ordered network regions across the cellular area. Among the four scores, the integrated 2D regularity score is designated as the optimized 2D regularity score.

### CLaSSiNet Workflow

We integrated the three classifier modules into a unified analysis pipeline, CLaSSiNet, to segment SMLM or EM images into four categories of cellular regions corresponding to four distinct network organizational states: 1D periodic network, 2D polygonal ordered network, disordered network, and non-network. The workflow proceeds sequentially as follows. (1) Connectivity Classifier Module—segments the SMLM image into network and non-network regions. (2) 1D Network Classifier Module—identifies 1D periodic networks within the network regions. (3) 2D Network Classifier Module—classifies the remaining network regions to detect 2D polygonal ordered networks. (4) Residual classification—remaining network regions are designated as disordered networks. This modular workflow allows systematic, hierarchical classification of network structures, generating spatial maps of four distinct organizational states across the cellular area.

### Actin Stress Fiber Detection

Stress fibers were identified from conventional fluorescence images of F-actin by using a dual-threshold segmentation approach. Images were normalized (0–255) and Gaussian-blurred (5 × 5 kernel) to reduce noise and then segmented using combined global (intensity >20) and local adaptive thresholding (111-pixel Gaussian window, offset = 2). Morphological closing (3 × 3 kernel) followed by opening (7 × 7 kernel) removed small gaps and noise. Connected regions were filtered to retain only elongated structures characteristic of stress fibers: eccentricity >0.8, area >250 μm^2^, and major axis length ≥2 μm. For subsequent angle-related analysis, the precise orientation of each identified fiber was determined and manually verified by using ImageJ.

### Confocal Image Cell Segmentation and Compartment Classification

The overall cell boundaries and nuclei were established to define the cellular region. The nuclei were segmented using the simple thresholding of the Hoechst stain. Whole cells were segmented using the watershed algorithm on membrane-stained images. Protein-enriched regions were robustly detected by combining adaptive local thresholding with global top-percentile filtering. This approach targets areas where protein signals are locally high while also being among the brightest global features. The resulting binary masks were further refined by morphological operations and size/intensity filtering (retaining regions >1000 pixels with an average intensity >60% of the maximum). This rigorous process allowed us to assign every pixel to one of five mutually exclusive compartments: blank region, cell body, cell edge, cell–cell junction, and nucleus.

## Supplementary Material



## Data Availability

All data and code needed to evaluate and reproduce the findings reported in this study are present in the main text, the Supporting Information, and/or the Zenodo repository. The source code for CLaSSiNet is publicly available on GitHub at https://github.com/thezhoulab/CLaSSiNet. The code and custom analysis scripts used to generate the figures are deposited in Zenodo^200^ (https://doi.org/10.5281/zenodo.18838318). MATLAB codes for image acquisition and STORM analysis are available on GitHub (https://github.com/ZhuangLab/) and archived in Zenodo^42^ (https://doi.org/10.5281/zenodo.3264857). The experimental data sets and manual labels used in this study are also available at Zenodo^201^ (https://doi.org/10.5281/zenodo.18750895).

## Notes

Detailed methodologies for SMLM data simulation, parameter optimization and robustness validations for all classifier modules, orthogonal verification using electron microscopy data, extended biological applications in diverse cell types, and comprehensive benchmarking against existing super-resolution analysis methods (PDF)
