# Supplementary material for: CLaSSiNet: A Computational Framework for High-Resolution Classification and Spatial Mapping of Heterogeneous Biological Network Architectures
Source: JACS Au. 2026 Mar 27;6(4):2637–55. doi: 10.1021/jacsau.5c01775 (PMC13126174; doi:10.1021/jacsau.5c01775)
Supplement: Supplementary file 1 [file au5c01775_si_001.pdf]

Supplementary Information for

CLaSSiNet: A Computational Framework for High-Resolution Classification and Spatial Mapping of Heterogeneous Biological Network Architectures

Yuan Tao[1,2,3], Ruobo Zhou[1,2,3,4,*]

[1]Department of Chemistry, The Pennsylvania State University, University Park, PA 16802, USA
[2]The Huck Institutes of Life Sciences, The Pennsylvania State University, University Park, PA 16802, USA
[3]Department of Biochemistry and Molecular Biology, The Pennsylvania State University, University Park, PA, 16802, USA
[4]Department of Biomedical Engineering, The Pennsylvania State University, University Park, PA 16802, USA

* To whom correspondence may be addressed. Email: ruobo.zhou@psu.edu

This Supplementary Information file includes:

Supplementary Figures: Figure S1 to S9

Supplementary Table: Table S1

References

Supplementary Figures

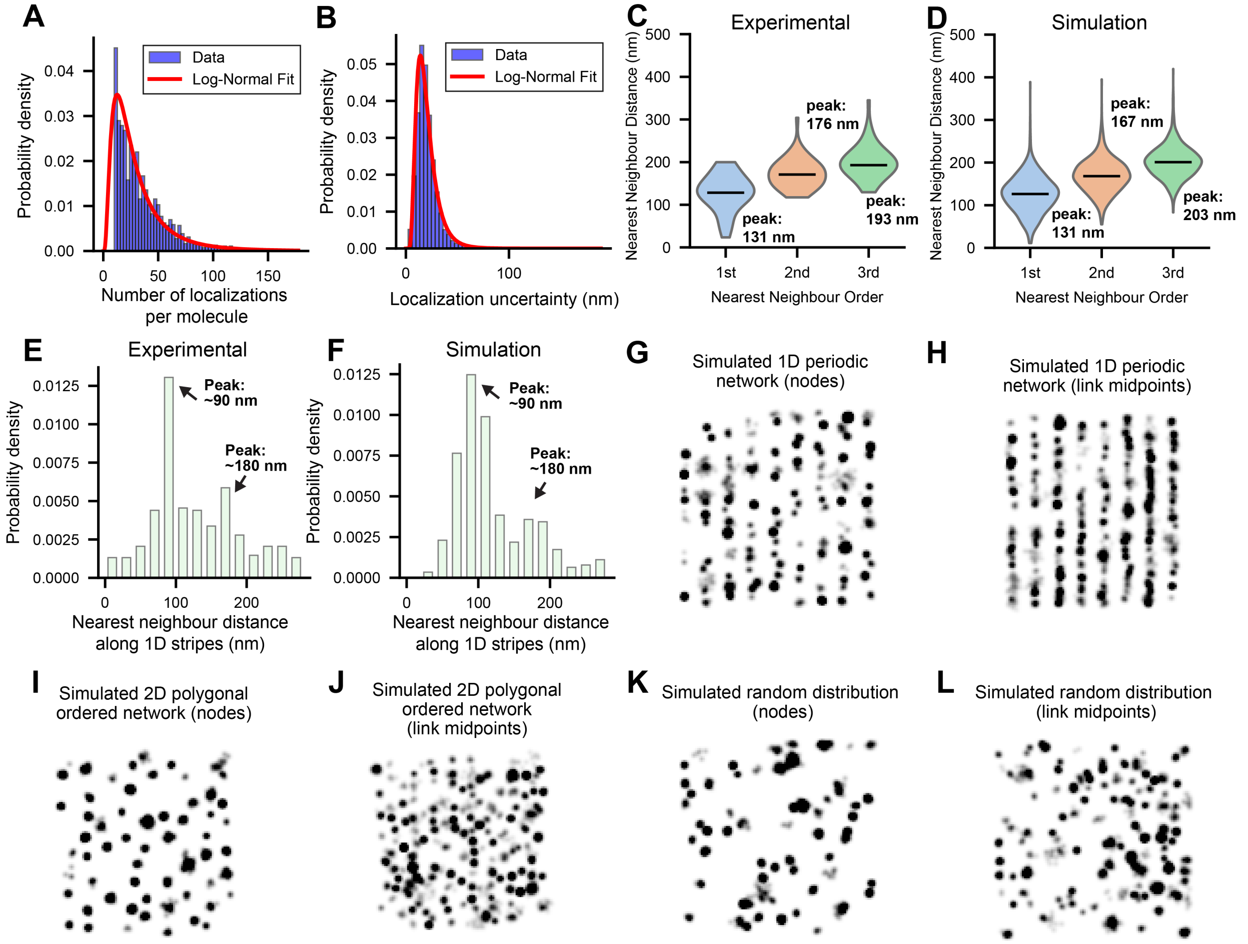

Figure S1. Parameter determination from experimental data for generating simulated SMLM images. (A, B) Distributions of the number of single-molecule localizations per molecular cluster (A) and localization uncertainties (i.e., the standard deviation of the Gaussian fit to single emitters) (B) obtained from experimental SMLM images of βIII-spectrin N-termini (labeling actin nodes) in neurons. Red solid lines represent Log-Normal probability density function (PDF) fits. These distributions were used to generate molecular clusters in simulated SMLM images that closely mimic experimental data. In the simulations, molecular clusters correspond to either nodes or the midpoints of links in the node-link network. **(C, D)** Violin plots of nearest-neighbor distance (NND) distributions for the first, second, and third nearest neighbors determined from the 2D polygonal ordered MPS network regions in the experimental SMLM images of actin nodes (i.e., N-terminus of βIII-spectrin immunolabeled) (C) and obtained from simulated SMLM node images (D). Center lines indicate the median. **(E, F)** Distributions of inter-cluster distances perpendicular to the 1D periodicity axis and of periods along the 1D periodicity axis, determined from the 1D periodic regions of experimental SMLM node images (E) and obtained from simulated SMLM

node images (F). Peak values of the distributions are indicated. **(G-J)** Representative simulated SMLM images generated using the parameters derived from panels (A-F), showing examples of node images (G) and link-midpoint images (H) for 1D periodic network, as well as examples of node images (I) and link-midpoint images (J) for 2D polygonal ordered network. **(K)** Same as (I) but for nodes with random distributions and equal node density as in (I). **(L)** Same as (J) but for link-midpoints with random distributions and equal link-midpoint density as in (J).

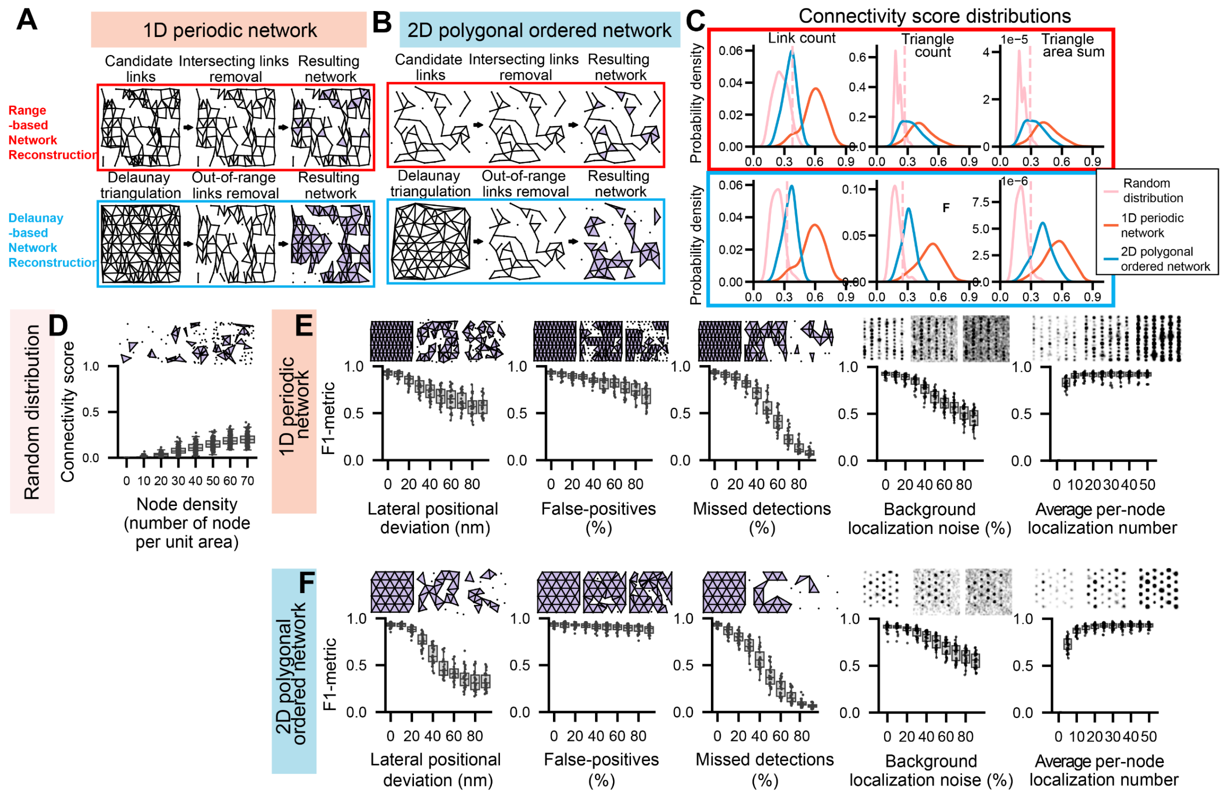

Figure S2. Optimization of the Connectivity Classifier Module. (A, B) Workflow for range-based (top) and Delaunay-based (bottom) network reconstruction, applied to simulated SMLM node images with 1D periodic (A) and 2D polygonal (B) distributions. **(C)** Kernel density estimation (KDE) plots showing the distributions of link count, triangle count, and triangle area sum calculated from simulated SMLM node images representing random, 1D periodic, and 2D polygonal networks, using range-based (top, in red box) and Delaunay-based (bottom, in blue box) network reconstruction. Dashed lines indicate the 95th percentile of the KDE distribution calculated from simulated SMLM node images with random node distributions, which is used as the threshold to segment network versus non-network regions. **(D)** Boxplots showing that connectivity scores shift to higher values as the node density increases in simulated random node distributions. A node-density-dependent threshold (defined as the 95th percentile of the KDE distribution from simulated random node distributions at each node density) is therefore applied for network segmentation. **(E, F)** Robustness analysis of the optimized Connectivity Classifier Module using simulated SMLM node images containing 1D periodic, and 2D polygonal networks, under five experimental noise conditions, including lateral positional deviation of nodes, added false-positive nodes (as a percentage of the total true nodes), missed node detections (as a percentage of the total true nodes), background localization noise (100% corresponds to the number of non-clustered background localizations equaling the total number of localizations across all nodes), and variation in per-node localization number (defined as the average number of localizations per node cluster). F1 metrics under these conditions are shown. Boxplots show the

median and interquartile range (first and third quartiles); whiskers denote the minimum and maximum values excluding outliers.

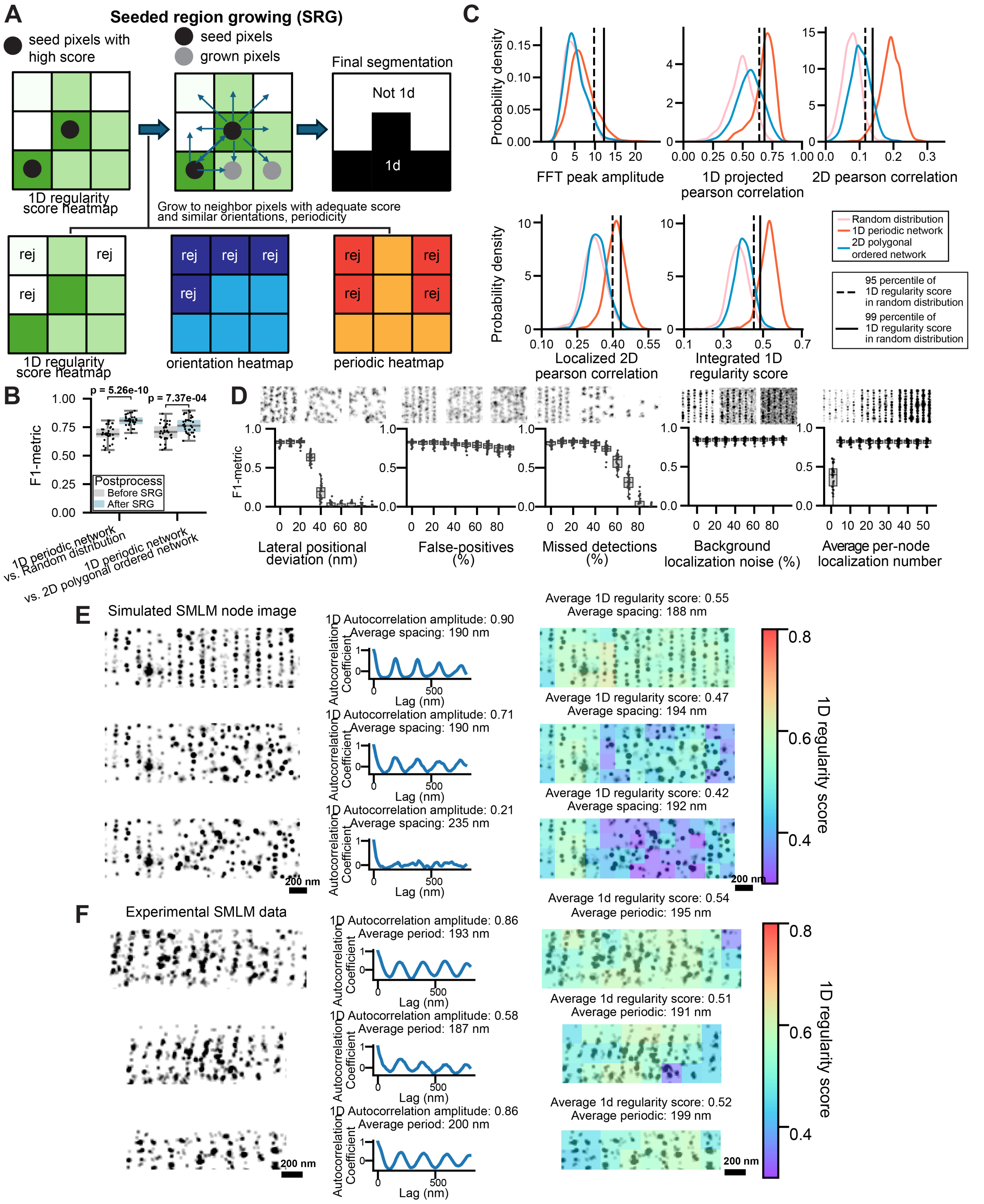

Figure S3. Optimization of the 1D Network Classifier Module. (A) Schematic illustrating the segmentation method based on a “seed region growing” (SRG) algorithm. A first threshold (99th percentile) was applied to the 1D regularity score heatmap to identify “seed” pixels with high 1D regularity scores, generating an initial segmentation of 1D network regions. Pixels adjacent to

these seeds that exceeded a second threshold of 1D regularity score (95th percentile) were then evaluated based on the orientation and period heatmaps: neighboring pixels were accepted or rejected depending on whether their orientation and period values were close to those of nearby seed pixels, thereby refining the segmentation boundary. **(B)** Boxplots of F1-metrics quantifying segmentation performance in distinguishing 1D periodic network regions from random or 2D polygonal network regions, before and after the "grow" refinement step in the seed-and-grow method. **(C)** Kernel density estimation (KDE) plots showing the distributions of FFT peak amplitude, 2D Pearson correlation, 1D projected Pearson correlation, localized 2D Pearson correlation, and integrated 1D regularity score, calculated from simulated SMLM node images representing random, 1D periodic, and 2D polygonal networks. Solid black lines and dashed lines indicate the 99th and 95th percentiles of the KDE distribution, respectively, calculated from simulated SMLM node images with random node distributions, which are used as the thresholds to segment 1D periodic network versus non-1D-network regions. **(D)** Robustness analysis of the optimized 1D Network Classifier Module using simulated SMLM node images containing 1D periodic networks under five experimental noise conditions, including lateral positional deviation of nodes, added false-positive nodes (as a percentage of the total true nodes), missed node detections (as a percentage of the total true nodes), background localization noise (100% corresponds to the number of non-clustered background localizations equaling the total number of localizations across all nodes), and variations in per-node localization number (defined as the average number of localizations per node cluster). **(E, F)** Comparison between 1D autocorrelation amplitudes determined by traditional 1D autocorrelation analysis and 1D regularity scores determined by the 1D Network Classifier Module, calculated from the same set of simulated SMLM images (E) or experimental SMLM images (F). F1-metrics under these conditions are shown. Scale bars: 200 nm. Boxplots show the median and interquartile range (first and third quartiles); whiskers denote the minimum and maximum values excluding outliers. *p*-values were calculated using a two-sided unpaired Student's *t*-test.

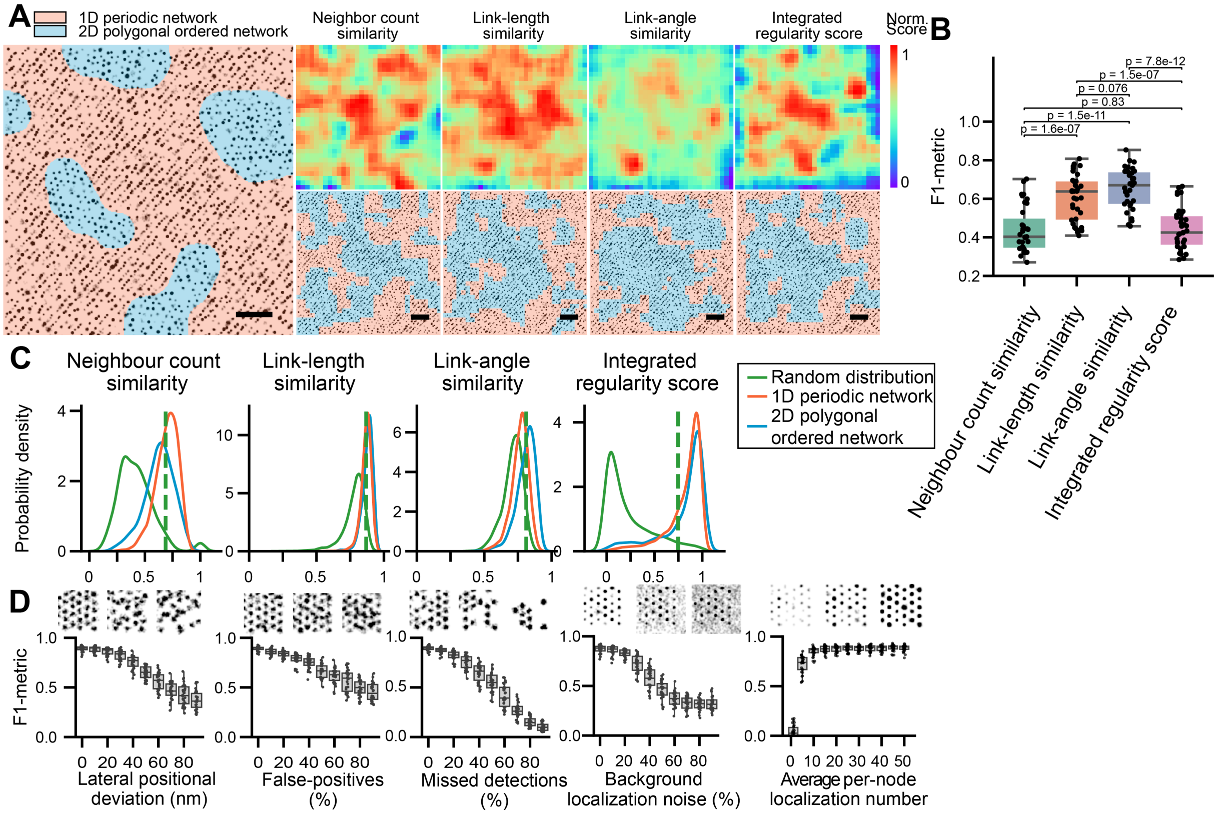

Figure S4. Optimization of the 2D Network Classifier Module. (A) Left: Representative simulated SMLM node image containing embedded islands of nodes arranged in 2D polygonal distribution, surrounded by nodes of equal density arranged in a 1D periodic network. Right: heatmaps (top) and segmented images (bottom) generated for the simulated SMLM image on the left, using the four candidate 2D regularity scoring methods. Scale bars: 1 µm. **(B)** Boxplots of F1-metrics quantifying the segmentation performance in distinguishing 2D polygonal network regions from 1D periodic network regions using the four candidate 2D regularity scoring methods. **(C)** Kernel density estimation (KDE) plots showing the distributions of the four candidate 2D regularity scores calculated from simulated SMLM node images representing random, 1D periodic, and 2D polygonal networks. Dashed lines indicate the 95th percentile of the KDE distribution calculated from simulated SMLM node images with random node distributions, which is used to segment 2D polygonal ordered network regions versus random distributions. This demonstrates that the LDA-based 2D regularity score provides the largest separation between the KDE distributions for 2D ordered and random networks, but less so between the KDE distributions for 2D polygonal ordered and 1D periodic ordered networks. **(D)** Robustness analysis of the optimized 2D Network Classifier Module using simulated SMLM node images containing 2D polygonal networks under five experimental noise conditions, including lateral positional deviation of nodes, added false-positive nodes (as a percentage of the total true nodes), missed node detections (as a percentage of the total true nodes), background localization noise (100% corresponds to the number of non-clustered background localizations equaling the total number of localizations across all nodes), and variation in per-node localization number (defined as the average number of

localizations per node cluster). F1 metrics under these conditions are shown. Boxplots show the median and interquartile range (first and third quartiles); whiskers denote the minimum and maximum values excluding outliers. *p*-values were calculated using a two-sided unpaired Student's *t*-test.

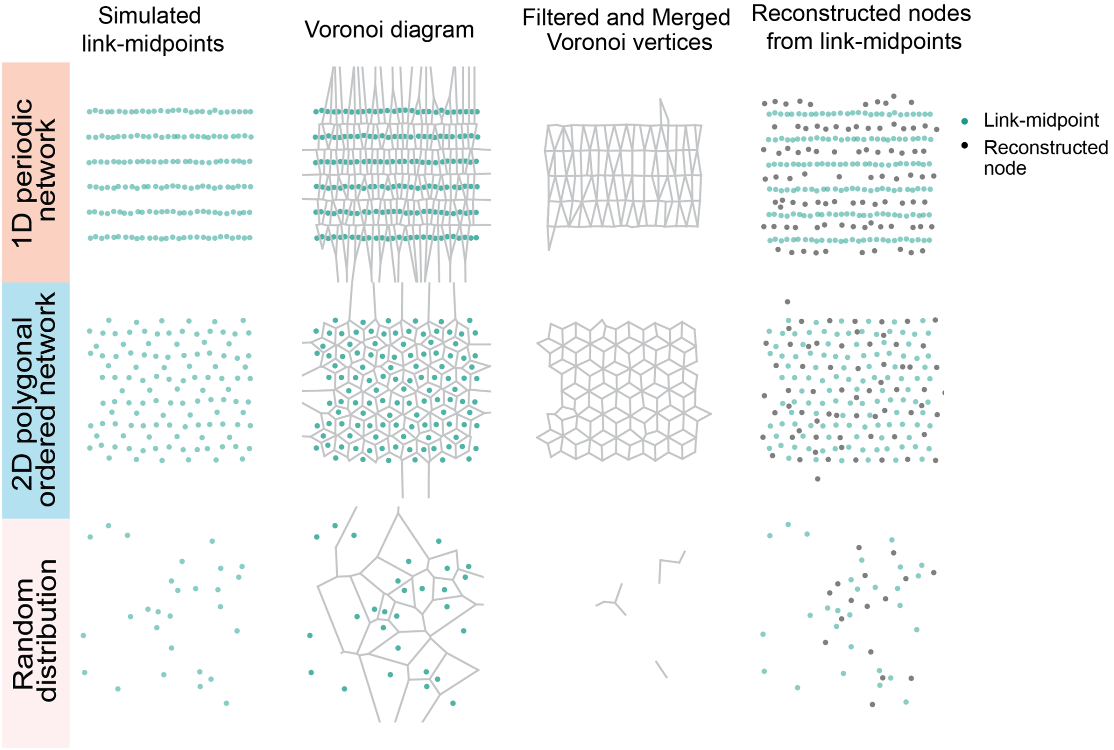

Figure S5. Voronoi tessellation-based algorithm to estimate node locations from link-midpoint distributions. Schematics illustrating the geometric principle of the algorithm designed to reconstruct N-terminal node positions from the simulated SMLM link-midpoint images for 1D periodic network, 2D polygonal ordered network, and random distributions, using a Voronoi tessellation-based algorithm.

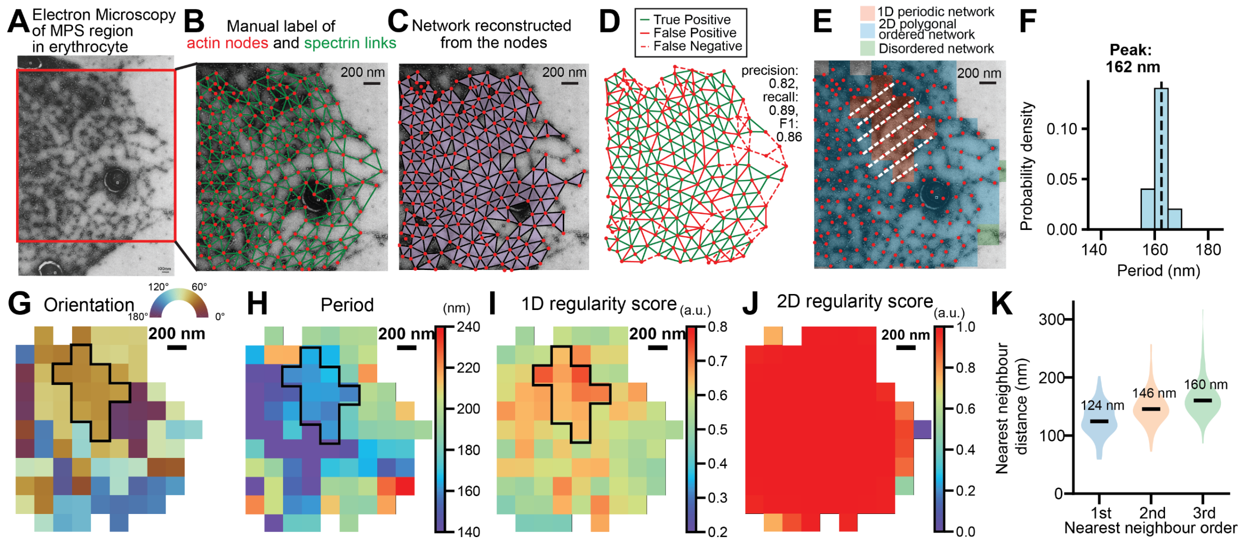

Figure S6. Orthogonal validation and cross-modality application of CLaSSiNet using an Electron Microscopy (EM) dataset. (A) Electron microscopy image of the membrane periodic skeleton (MPS) in an erythrocyte. **(B)** Ground truth annotation of the EM image in (A), with manually labeled actin nodes (red dots) and spectrin links (green lines). **(C)** Network reconstruction generated by CLaSSiNet using the actin node coordinates from (B) as input. **(D)** Overlay comparison between the CLaSSiNet-reconstructed network (prediction) and the annotated ground truth. Green solid lines indicate True Positives (matching links); red solid lines indicate False Positives (spurious links); red dashed lines indicate False Negatives (missed links). **(E)** CLaSSiNet segmentation map showing the identified MPS organizational states. White dashed lines indicate the local axis of 1D periodicity. **(F)** Distribution of 1D periods obtained from the segmented 1D periodic regions identified in (E), showing a peak at ~162 nm. **(G–J)** Orientation heatmap (G), period heatmap (H), 1D regularity score heatmap (I), and 2D regularity score heatmap (J) generated for the EM image in (E). **(K)** Violin plots of the first, second, and third nearest-neighbor distances (NNDs) within the identified 2D polygonal ordered network regions in (E). Center lines indicate the median. Scale bars: 200 nm. The EM image shown in Panels A-C is adapted with permission from Supplementary Reference [1]. Copyright 1987 Rockefeller University Press.

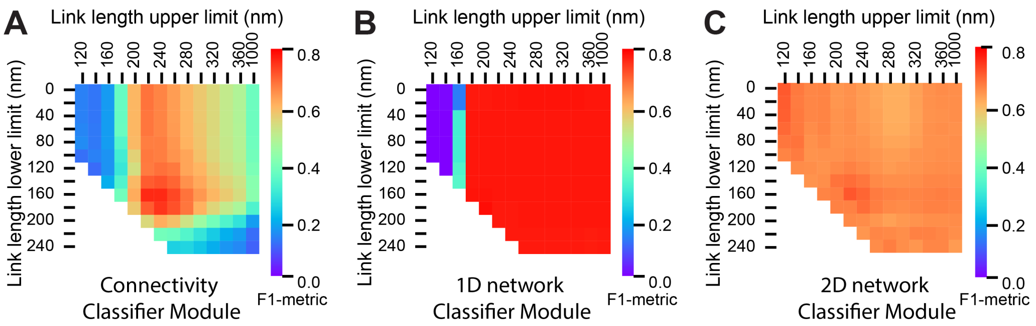

Figure S7. Sensitivity analysis of CLaSSiNet classifier performance to the allowable link-length range. (**A**) Heatmap showing the F1 metric of the Connectivity Classifier Module as a function of the allowable link-length range, defined by the upper (x-axis) and lower (y-axis) bounds, using simulated node datasets containing ordered network regions embedded in a randomly distributed background. The color scale indicates the classification performance (*i.e.*, F1-metric). (**B**) Corresponding F1 metric heatmap for the 1D Network Classifier Module. (**C**) Corresponding F1 metric heatmap for the 2D Network Classifier Module.

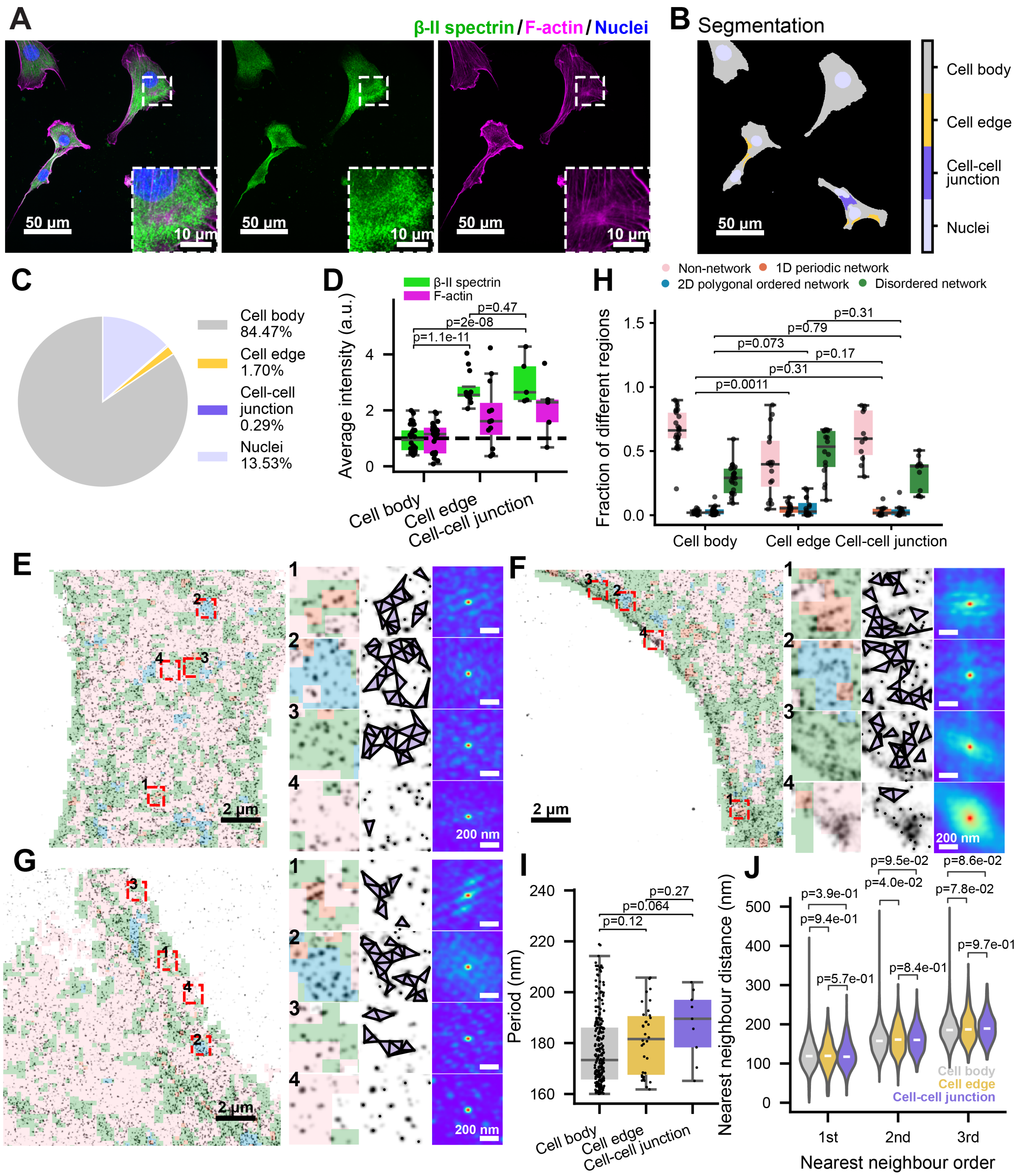

Figure S8. CLaSSiNet reveals distinct MPS organizations across three subcellular zones in fibroblasts. (A) Representative three-color confocal images of 3T3 cells co-stained for nuclei (blue, Hoechst), βII-spectrin (green, βII-spectrin antibody), and F-actin (magenta, phalloidin). Scale bar: 50 μm. Inset: Magnified view of the boxed region. Scale bar: 10 μm. **(B)** Segmented image corresponding to (A), showing four subcellular zones: nucleus, cell body, cell edge, and cell–cell junction. Scale bar: 50 μm. **(C)** Quantified area fractions of the four subcellular zones. **(D)** Boxplots showing average fluorescence intensities of βII-spectrin and F-actin across the three

non-nuclear subcellular zones (cell body, cell edge, and cell–cell junction). **(E–G)** Left: Representative SMLM (STORM) images of βII-spectrin in three subcellular zones, cell body (E), cell edge (F), and cell–cell junction (G), overlaid with CLaSSiNet segmentation into four MPS organizational states: 1D periodic network, 2D polygonal ordered network, disordered network, and non-network regions. Scale bar: 2 μm. Middle: Magnified views of boxed regions enriched in each of the four organizational states. Right: Reconstructed node-link networks and corresponding 2D autocorrelation maps confirming the expected MPS organizational patterns. Scale bar: 200 nm. **(H)** Boxplots showing the area fractions of the four MPS organizational states across the three subcellular zones. **(I)** Boxplots of 1D periodicity periods measured from 1D periodic network regions across the three subcellular zones. Boxplots show the median and interquartile range (first and third quartiles); whiskers denote the minimum and maximum values excluding outliers. **(J)** Violin plots comparing the first, second, and third nearest-neighbor distance (NND) distributions of 2D polygonal ordered network regions across the three subcellular zones. Center lines indicate the median. p-values were calculated using a two-sided unpaired Student's t-test.

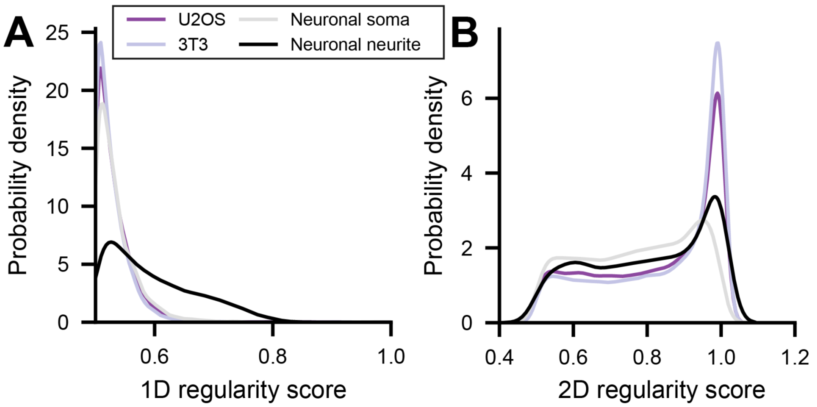

Figure S9. Distributions of 1D and 2D regularity scores for U2OS cells, 3T3 cells, neuronal somas, and neuronal neurites. (A, B) Kernel density estimation (KDE) plots showing the distributions of the 1D regularity score (A) and the 2D structural regularity score (B) for all network regions across U2OS cells, 3T3 cells, neuronal somas, and neuronal neurites.

Supplementary Table

Method Name	1D Period Detection	1D Orientation Detection	2D Regularity Detection	Network Reconstruction	Multi-State Classification*	Automatic ROI Selection	Spatially Resolved Heatmaps	References
CLaSSiNet (Our Method)	✓	✓	✓	✓	✓	✓	✓	This work
SReD	◐[a]	◐[b]	✗	✗	✗	✓	✓	Mendes et al., 2025[2]
Napari-WaveBreaker	✓	✓	✗	✗	✗	✗	✗	Vanspauwen et al., 2025[3]
Gollum	✗	◐[b]	✗	✗	✗	✓	✗	Barabas et al.,2017[4]
1D Autocorrelation	✓	✗	✗	✗	✗	✗	✗	Xu et al., 2013[5]
Sinusoid Fitting	✓	✗	✗	✗	✗	✗	✗	Leterrier et al. 2015[6]
2D Network Reconstruction	✗	✗	✗	✓	✗	✗	✗	Han et al., 2017[7]
2D Autocorrelation	✗	✗	✓	✗	✗	✗	✗	Han et al., 2017[7]

Table S1. Feature-by-feature comparison of CLaSSiNet with existing super-resolution network analysis methods.

✓ = Yes, ✗ = No, ◐ = Partial

Footnotes:

[*] Capable of distinguishing four distinct classes: 1D periodic network, 2D polygonal ordered network, disordered network, and non-network.

[a] Outputs a single globally averaged periodicity value for the entire field of view, thereby failing to capture region-to-region variance within the sample.

[b] Orientation is derived from overall neurite morphology rather than local network topology; consequently, it cannot resolve 1D alignment in cellular regions lacking neurite-like geometry.

References

1. Liu, S. C., Derick, L. H. & Palek, J. Visualization of the hexagonal lattice in the erythrocyte membrane skeleton. *J. Cell Biol.* **104**, 527–536 (1987).
2. Mendes, A. *et al.* Structural Repetition Detector for multi-scale quantitative mapping of molecular complexes through microscopy. *Nat. Commun.* **16**, 5767 (2025).
3. Vanspauwen, S. K., Luque-Fernández, V. & Rasmussen, H. B. A correlation-based tool for quantifying membrane periodic skeleton associated periodicity. *Front. Neuroinformatics* **19**, (2025).
4. Barabas, F. M. *et al.* Automated quantification of protein periodic nanostructures in fluorescence nanoscopy images: abundance and regularity of neuronal spectrin membrane-associated skeleton. *Sci. Rep.* **7**, 16029 (2017).
5. Xu, K., Zhong, G. & Zhuang, X. Actin, Spectrin, and Associated Proteins Form a Periodic Cytoskeletal Structure in Axons. *Science* **339**, 452–456 (2013).
6. Leterrier, C. *et al.* Nanoscale Architecture of the Axon Initial Segment Reveals an Organized and Robust Scaffold. *Cell Rep.* **13**, 2781–2793 (2015).
7. Han, B., Zhou, R., Xia, C. & Zhuang, X. Structural organization of the actin-spectrin–based membrane skeleton in dendrites and soma of neurons. *Proc. Natl. Acad. Sci.* **114**, E6678–E6685 (2017).
